# Biodiversity of entomopathogenic fungi in soils of eastern China

**DOI:** 10.1128/spectrum.02904-25

**Published:** 2026-02-13

**Authors:** Wei Chen, Tongyi Liu, Ke Zhang, Xiangyu Hu, Xue Guan, Qiongbo Hu, Qunfang Weng

**Affiliations:** 1 College of Plant Protection, State Key Lab of Green Pesticide, South China Agricultural University 12526, Guangzhou, China; 2 Ganzhou Polytechnic, Ganzhou, Jiangxi, China; Agroscope, Nyon, Switzerland

**Keywords:** entomopathogen, fungi, *Purpureocillium lilacinum*, *Gongronella*, *Yunnania*, *Sarocladium*

## Abstract

Sustainable agriculture requires the development of biological control technologies. Entomopathogenic fungi (EPF) are important resources of microbial control agents, yet their diversity in soil with various habitats remains poorly understood. In this study, we systematically investigated the EPF diversity in soils of eastern China. A total of 455 strains of 42 EPF species belonging to 18 genera were isolated and identified from 540 soil samples collected from 108 sites representing five habitats (crop, orchard, forest, fallow, and grasslands) in Anhui, Jiangsu, Jiangxi, Shandong, Shanghai, and Zhejiang regions. Habitats had a greater influence on EPF diversity than regions. Overall, EPF diversity in natural habitats (forests and grasslands) was slightly higher than in agricultural habitats (crops, orchards, and fallow lands), particularly in terms of species richness. Among agricultural habitats, orchard had the highest evenness and relatively low species richness, while fallow land showed the lowest diversity, which may be related to management intensity or vegetation cover. Furthermore, the results also demonstrated the similarity of the EPF community structures among different habitats or regions; *Purpureocillium lilacinum* was the dominant species, while *Penicillium citrinum* and *Metarhizium anisopliae* were the minor dominant species in all habitats. In contrast, *Metarhizium robertsii*, *Penicillium janthinellum*, *Marquandomyces marquandii*, and *Trichoderma spirale* exhibited variable dominance depending on the habitat. This research provides new insights to understand the fungal diversity.

**IMPORTANCE:**

We recognized a total of 455 strains of 42 entomopathogenic fungi (EPF) species in 18 genera, including 7 putative new species with substantial insecticidal activity, which have potential for application as biological control agents. The discovery will enhance the understanding that agricultural activity interferes with the EPF distribution. Furthermore, the results highlight the conservation and application of EPF.

## INTRODUCTION

Population growth and environmental pollution call for sustainable agricultural production, where pest biocontrol plays a significant role in the substitution of chemical pesticides and food security assurance. Entomopathogenic fungi (EPF) are a key force to regulate insect populations in nature. There are more than 1,000 species of EPF discovered to date in the world (1). The highly bioactive EPF, such as *Beauveria bassiana*, *Metarhizium anisopliae*, and *Purpureocillium lilacinum*, have been widely employed as biological control agents in agricultural pest management and are registered in many countries as mycopesticides (2). Meanwhile, many EPF such as *Ophiocordyceps sinensis* and *Cordyceps cicadae* are expensive traditional medicines used in East Asia (3). Nevertheless, numerous EPF serve as opportunistic entomopathogens, while their ecological roles and potential utilization remain frequently neglected.

Compared with chemical pesticides, EPF exhibit lower environmental toxicity, higher biodegradability, and stronger host specificity, thereby offering a sustainable option in pest control. As global concerns over pesticide resistance continue to escalate, biological control approaches have garnered significant attention, and EPF have emerged as promising biocontrol agents. However, EPF still face challenges such as insufficient resources and suboptimal efficacy, which hinder their development (4).

EPF infect their insect hosts by penetrating with special structures such as appressoria and germ tubes, which is similar to the “contact toxicity” of chemical insecticides and enables EPF to control piercing-sucking pests such as aphids and whiteflies. This is a characteristic that bacterial and viral insecticides do not possess (2). Actually, EPF are not merely entomopathogens killing insects; they play important roles in natural ecosystems through multi-level trophic interactions, such as saprophytes, plant endophytes, and parasites (5). For example, the wheat endophytic *B. bassiana* and *M. robertsii* alter the plants' secondary metabolites, thereby inhibiting the population growth of insect pests *Rhopalosiphum padi* and *Aphis fabae* (6), while the interactions of *Syncephalastrum* sp. (Mucorales) with fungus garden and leaf-cutter ants offer new opportunities in their integrated pest management (7). However, more intriguing mysteries surrounding EPF’s biotic-soil interactions remain to be uncovered.

EPF are often soil inhabitants, and large numbers of EPF can persist for a long time in soil; sometimes, they can also reproduce in the rhizosphere of plants, thereby maintaining a certain population level in the soil (8). After infecting insects, EPF can enter a dormant state in the soil to survive adverse conditions, such as low temperatures or reduced soil-water activity. Many EPF species are effective in controlling soil pests; for example, granular formulations of *B. bassiana* against *Frankliniella occidentalis* on eggplants resulted in a nearly 70% control efficacy (9). Furthermore, soil is a key source for EPF genetic resources. In recent years, many new EPF species have been isolated and identified from soil (10). However, economic and social development has also brought about numerous soil issues, such as soil pollution (heavy metals, chemicals, etc.), vegetation degradation, and decreased soil organic matter (11). Undoubtedly, these problems will inevitably impact the diversity of EPF in the soil.

Therefore, we initiated a large-scale study on the diversity of EPF in soils across various regions of China. The purpose was to understand the relationship between EPF diversity and soil habitats as well as geographical regions and to discover new fungal resources, and thereby promoting the development of biological control technologies (8, 12, 13). In this study, we investigated EPF in soils of eastern China by isolating and identifying EPF strains, acquiring novel germplasm resources, and elucidating their diversity characteristics across different habitats and regions. The eastern region of China is the most economically developed area in the country, with a large population, well-developed industry, and agriculture. Geographically, it has more plains and fewer high mountains, with numerous rivers and lakes, and its climate ranges from subtropical to temperate. Understanding the diversity of soil EPF in this area will promote the development of biopesticides and sustainable agriculture.

## RESULTS

### Numbers of EPF identified

From the 540 soil samples (Fig. S1), 444 strains belonging to 35 species of 15 genera were identified based on the morphological and molecular phylogenetic analysis of internal transcribed spacer (ITS) and rRNA large subunit (LSU) (Fig. S2 and S3; Table S1), and they were recognized as EPF species according to the published documents (Table 1). Additionally, the other 26 strains belonging to putative new species were subject to bioassay and resulted in the discovery of 7 putative new EPF species with 11 strains for which the mortalities of *Ostrinia furnacalis* were significantly higher (*P* < 0.05) than for the control group (Table 2). In total, 455 EPF strains of 42 species in 18 genera were isolated and identified in this research (Table 1; Table S1). All sequences generated in this study were deposited in the GenBank database. Phylogenetic trees generated from LSU and ITS sequences showed similar topologies; therefore, only the ITS-based phylogeny is presented in this manuscript (Fig. S3).

**TABLE 1:** Information on EPF strains

Species	Family	Strain	Ref.	Num.^*b*^	
Hypocreales, Pezizomycotina, Ascomycota					**323**
*Clonostachys chloroleuca*	Bionectriaceae	AH16A03, AH20A02, JS11B01, JX11B01, JX11D03, JX11E05, JX18A03, SH02D04	14	8	8
*Marquandomyces marquandii*	Clavicipitaceae	AH10B03, AH18A02, JS01A04, JS09C02, JS15D02, JX11C01, SD11E03, SH01A05, SH04B03, SH04C03, SH05D01, SH07B03, ZJ12B03	15	13	
*Metapochonia bulbillosa*	Clavicipitaceae	AH18C01	16	1	
*M. anisopliae*	Clavicipitaceae	AH02C03, AH09E01, AH09E03, AH16C02, AH16E02, AH17C03, AH17D02, AH19B03, AH19B04, JS03D03, JS10B02, JS11E01, JS11E04, JS13B03, JS15D01, JX13A06, JX17E01, SD04A03, SD05D03, SD10E03, SD13A02, SD13C03, SD13E03, SD15A01, SD17A02, SH05A01, ZJ02D01, ZJ14A04, ZJ14B03		29	
*M. baoshanense*	Clavicipitaceae	SD05B01, SD17B04, SH02A04	17	3	
*M. brunneum*	Clavicipitaceae	AH07D05, JS13E03, SH05C01		3	
*M. gaoligongense*	Clavicipitaceae	AH07D02, AH17E01, JS01E02	17	3	
*M. robertsii*	Clavicipitaceae	JS01A06, JS02A01, JS13B04, JS13E01, JS13E02, JS14A05, JS19A01, JS19C04, JX11D04, JX15B05, SD11A05, SD11A07, SD12A05, SD12C03, SD13B05, SD14B02, SD17E02, SH02B03		18	
*Metacordyceps chlamydosporia*	Clavicipitaceae	ZJ01C02	18	1	71
*B. bassiana*	Cordycipitaceae	AH07D06, AH08C02, JS02B02, JS05B02, JS12C01, JS19E03, SD05A04, SD11E02		8	
*B*. sp1^*a*^	Cordycipitaceae	AH08D02	This study	1	
*B*. sp2^*a*^	Cordycipitaceae	JS02A02, SD18B02	This study	2	
*Lecanicillium anqingense*	Cordycipitaceae	AH13B2, AH08C5	10	2	
*L. renii*	Cordycipitaceae	JX15A210, ZJ08C4	10	2	
*Simplicillium subtropicum*	Cordycipitaceae	JS14A01	19	1	16
*Trichoderma asperellum*	Hypocreaceae	AH13E02, JS16A06, JX14A04, SH06E03, ZJ09A04, ZJ11D06	20	6	
*T. citrinoviride*	Hypocreaceae	JS07C03	21	1	
*T. spirale*	Hypocreaceae	AH14C02, AH14E02, JX15E05, JX17B02, JX20D03, ZJ06C03, ZJ07A01, ZJ09B01, ZJ11B03, ZJ16B01, ZJ19B01	22	11	18
*Fusarium solani*	Nectriaceae	JX19D01, SD11A04, ZJ05D02, ZJ16E02, ZJ19C02	23	5	5
*Purpureocillium jiangxiense*	Ophiocordycipitaceae	JX13B01	24	1	
*P. lavendulum*	Ophiocordycipitaceae	AH01A01, AH02C02, JS13B05, JS17C02, SD04C03, SD16B05, SH07B02, ZJ04B02, ZJ12C01, ZJ12D01	25	10	
*P. lilacinum*	Ophiocordycipitaceae	AH01B03, AH01B04, AH02B02, AH03B02, AH04A02, AH05E01, AH05E03, AH06D02, AH08B03, AH09E02, AH10D03, AH10E02, AH11D01, AH11E03, AH12B04, AH12C02, AH13C02, AH13C03, AH14C01, AH15C01, AH16D01, AH16E03, AH17C04, AH18C02, AH18D02, AH18E03, AH19B02, AH19C02, AH20A01, AH20A04, AH20B01, AH20B02, AH20D03, JS01A03, JS02B01, JS02C02, JS03D01, JS03D02, JS04A01, JS05A04, JS05B01, JS06C02, JS06E01, JS07C04, JS08B02, JS09D01, JS09D02, JS10B01, JS10B04, JS10D02, JS11A04, JS11C02, JS11E02, JS12C02, JS12D01, JS12E02, JS13B01, JS13B02, JS14B01, JS14D01, JS15C01, JS15C02, JS16A04, JS17B01, JS17C01, JS19E01, JS20E02, JX02B01, JX02B02, JX03C01, JX03C02, JX03D01, JX04A04, JX04A07, JX04B01, JX05A02, JX06C03, JX06C05, JX08E02, JX08E03, JX09A03, JX09A04, JX11A04, JX12C06, JX12E01, JX13A03, JX13A04, JX14A05, JX15A02, JX15B01, JX15C01, JX15E04, JX16C02, JX16C04, JX17B03, JX17D05, JX17E02, JX18B02, JX19A01, JX19B01, JX19D02, JX20A05, JX20C04, SD01A01, SD01B01, SD01C01, SD01E01, SD01E02, SD01E03, SD03B01, SD03B02, SD04B02, SD04C04, SD05A01, SD05A03, SD06A01, SD06C01, SD06D05, SD08B01, SD08D02, SD08D03, SD09A03, SD09C01, SD09E01, SD10E02, SD11A06, SD11B01, SD11E04, SD12B02, SD12B03, SD12E02, SD13B02, SD13C04, SD13D01, SD13D03, SD13E04, SD14A02, SD14B01, SD14B03, SD14E02, SD15D01, SD15D04, SD15E01, SD16B02, SD16B03, SD16B07, SD16E01, SD17A01, SD17B02, SD17C01, SD17D02, SD17E01, SD18C02, SD18C03, SD19E01, SD20D01, SD20E02, SD20E03, SD20E04, SH01A01, SH02A01, SH03A03, SH03B03, SH05B01, SH05B03, SH06B02, SH06B04, SH07C03, ZJ01B01, ZJ01E02, ZJ02A02, ZJ02C02, ZJ03A02, ZJ04A02, ZJ06C01, ZJ08C01, ZJ10C02, ZJ10E03, ZJ11D02, ZJ12A04, ZJ12C02, ZJ13D06, ZJ14A03, ZJ15A02, ZJ16A01, ZJ16D04, ZJ17A04, ZJ18A05, ZJ19C05, ZJ20E01		190	201
*Sarocladium* sp1^*a*^	Sarocladiaceae	JS06C01, JS07C02	This study	2	
*S*. sp2^*a*^	Sarocladiaceae	JX12E02, ZJ19A01	This study	2	4
Microascales, Pezizomycotina					**1**
*Yunnania* sp.^*a*^	Microascaceae	JX11B02	This study	1	
Sordariales, Pezizomycotina					**8**
*Chaetomium cochliodes*	Chaetomiaceae	AH05E02, AH11E01, JS07A02, JX12D02, SD11A02, SD15C04	26	6	
*C. globosum*	Chaetomiaceae	AH10D04, JS19D03	27	2	
Eurotiales, Pezizomycotina					**112**
*Aspergillus aureoterreus*	Aspergillaceae	JX06C06, JX13E04, JX18E04	28	3	
*A. flavus*	Aspergillaceae	AH06D03, AH08B02, JS11E03, JX11D01, JX13A05, SD02B01, SD19A02, SH04A03, ZJ05A03	29	9	
*A. fumigatus*	Aspergillaceae	JS02B04, ZJ20C03	30	2	
*A. insuetus*	Aspergillaceae	AH07B01, JS02C01, JS02D03, JS06D01	31	4	
*A. ochraceus*	Aspergillaceae	AH01D03, AH16D04, ZJ15D02	32	3	
*Penicillium brefeldianum*	Aspergillaceae	JS08B03, JX15A03, JX15A06, JX16B03, ZJ01E03, ZJ05C02	33	6	
*P. citrinum*	Aspergillaceae	AH01D02, AH01D04, AH11D02, ZJ12B01, JS07A01, SD20B01, JS16A05, SD18E02, JX15E03, SD01A02, ZJ06A02, ZJ11C03, SD16E02, AH08C01, SH04D01, AH20C01, SD02B04, ZJ09A02, ZJ12C03, SH07A05, SD04A01, JS12B02, AH13A02, JX15D01, JS04C03, AH07E01, JX11A02, SD03D01, AH06D04, JS06D02, ZJ13D01, AH02E01, ZJ16A03, ZJ21D01, JX14A03, JX14B03, JX17D06, SH01C02, JS02B05, AH12A02, ZJ01A01, JS04B02, AH03B01, JS20A01, AH02A01, ZJ15E02, AH05B03, SH06D02, JX11A01, SH07A03, ZJ13A01, ZJ03B01, JX15C02, JX16E03, SD09C02, AH13D01, SD12B01, AH06C03, SD11A03, SH03B05, ZJ04B01, ZJ02B03, AH02E01, AH05B02, JX20A03	34	65	
*P. daleae*	Aspergillaceae	AH14E01, ZJ14B01, ZJ17B03, ZJ18D01	35	4	
*P. guanacastense*	Aspergillaceae	AH03B03, AH12B01, SD02B02, SD03D02	36	4	
*P. janthinellum*	Aspergillaceae	JX06C01, JX18A01, JX19A02, ZJ03C01, ZJ03C03, ZJ08A02, ZJ08E02, ZJ09A05, ZJ09D11, ZJ18C01	37	10	
*P. oxalicum*	Aspergillaceae	SH07C02	38	1	
*P. sizovae*	Aspergillaceae	SD14C01	39	1	
Trichosphaeriales, Pezizomycotina					**8**
*Plectosphaerella cucumerina*	Trichosphaeriaceae	AH08D03, AH20E01, AH20E05, SD20B04, SD20E01, SH01D01, SH02A02, SH04C05	40	8	
Mucorales, Mucoromycotina, Mucoromycota					**3**
*Gongronella* sp1^*a*^	Cunninghamellaceae	JX20B02	This study	1	
*G*. sp2^*a*^	Cunninghamellaceae	JX09A02, JX15B03	This study	2	
Strain counts				**455**	

^*a*^ The putative new species this study found.

^*b*^ The bold values indicated that numbers of taxa above the species.

**TABLE 2:** Bioactivity of putative new species against third-instar larvae of Asian corn borer (ACB)

Putative novel species	Strain	Cumulative mortality (%) (mean ± SE)^*a*^				
		5 d	7 d	9 d	11 d	13 d
*Beauveria* sp1	AH08D02	90.00 a	100.00 a	100.00 a	100.00 a	100.00 a
*Beauveria* sp2	JS02A02	0	36.67 ± 6.67 c	73.33 ± 6.67 b	100.00 a	100.00 a
	SD18B02	30.00 ± 5.77 b	66.67 ± 6.67 b	100.00 a	100.00 a	100.00 a
*Gongronella* sp1	JX20B02	23.33 ± 3.33 b	46.67 ± 5.77 c	50.00 ± 5.77 c	50.00 ± 6.67 b	56.67 ± 6.67 b
*Gongronella* sp2	JX09A02	0	3.33 ± 3.33 e	3.33 ± 3.33 e	26.67 ± 8.82 c	60.00 ± 10.00 b
	JX15B03	13.33 ± 3.33 c	33.33 ± 6.67 c	40.00 cd	50.00 ± 5.77 b	56.67 ± 6.67 b
*Sarocladium* sp1	JS06C01	0	3.33 ± 3.33 e	23.33 ± 8.82 d	40.00 ± 5.77 bc	49.33 ± 5.77 bc
	JS07C02	0	0	3.33 ± 3.33 e	23.33 ± 6.67 cd	40.00 ± 5.77 bc
*Sarocladium* sp2	ZJ19A01	0	0	0	16.67 ± 6.67 cd	33.33 ± 8.82 c
	JX12E02	3.33 ± 3.33 d	23.33 ± 6.67 d	32.00 ± 5.77 d	70.00 ± 5.77 b	90.00 ± 6.67 a
*Yunnania* sp.	JX11B02	0	3.33 ± 3.33 e	6.67 ± 3.33 e	10.00 ± 5.77 d	23.33 ± 5.77 d
*Cunninghamella* sp.	JS20A03	0	0	0	10.00 ± 5.77 d	16.67 ± 5.77 d
*Cunninghamella* sp.	JS05A03	0	0	3.33 ± 3.33 e	6.67 ± 3.33 d	16.67 ± 6.67 d
*Cunninghamella* sp.	JS09A02	0	3.33 ± 3.33 e	3.33 ± 3.33 e	6.67 ± 3.33 d	6.67 ± 3.33 e
*Cunninghamella* sp.	JX18D04	0	3.33 ± 3.33 e	3.33 ± 3.33 e	3.33 ± 3.33 d	10.00 ± 5.77 d
*Cunninghamella* sp.	SH05B02	6.67 ± 6.67 cd	6.67 ± 3.33 e	13.33 ± 3.33 e	13.33 ± 3.33 d	13.33 ± 3.33 d
*Cunninghamella* sp.	AH02B01	0	0	3.33 ± 3.33 e	10.00 ± 5.77 d	16.67 ± 3.33 d
*Cunninghamella* sp.	JX11A03	0	0	0	3.33 ± 3.33 d	10.00 ± 5.77 d
*Cunninghamella* sp.	ZJ18A02	0	3.33 ± 3.33 e	3.33 ± 3.33 e	10.00 ± 5.77 d	13.33 ± 6.67 d
*Cunninghamella* sp.	AH14B01	0	6.67 ± 3.33 e	10.00 ± 5.77 e	13.33 ± 5.77 d	16.67 ± 6.67 d
*Cunninghamella* sp.	AH17A01	0	0	0	0	6.67 ± 3.33 e
*Cunninghamella* sp.	JX17D01	10.00 ± 5.77 c	10.00 ± 5.77 e	13.33 ± 3.33 e	13.33 ± 3.33 d	16.67 ± 3.33 d
*Cunninghamella* sp.	SD18C04	3.33 ± 3.33d	3.33 ± 3.33 e	3.33 ± 3.33 e	6.67 ± 3.33 d	6.67 ± 3.33 e
*Keithomyces* sp.	SD04A02	0	0	0	0	10.00 ± 5.77 d
*Keithomyces* sp.	JX11E06	0	0	3.33 ± 3.33 e	10.00 ± 5.77 d	16.67 ± 5.77 d
*Marquandomyces* sp.	AH20E02	0	3.33 ± 3.33 e	3.33 ± 3.33 e	3.33 ± 3.33 d	3.33 ± 3.33 e
CK		0	0	0	0	3.33 ± 3.33 e

^*a*^ The different letters behind the mean ± SE indicate the significant difference (*P* < 0.05, Duncan’s multiple range test [DMRT]).

### EPF isolation rates in different habitats and regions

There were 455 EPF strains isolated from 269 soil samples (total 540), with an isolation rate of 49.81% (Table 3). The EPF isolation rates varied among different habitats: forest had the highest isolation rate of 60.19%, while the other habitats (crop, orchard, fallow, and grasslands) followed with 39%–60% isolation rates. In addition, the EPF isolation rates within regions varied. The Jiangxi and Anhui regions had the lowest and highest isolation rates of 43% and 55%, respectively, and for the other regions, they ranged from 47% to 54% (Table 3).

**TABLE 3:** EPF isolation of soil samples from different habitats and regions

Habitat	Sample number	EPF(+) samples^*a*^	EPF isolation rate (%)	Region	Sample number	EPF(+) samples^*a*^	EPF isolation rate (%)
Crop	108	64	59.26	Anhui	100	76	55.00
Forest	108	65	60.19	Jiangsu	100	60	48.00
Grassland	108	52	48.15	Jiangxi	100	53	43.00
Orchard	108	43	39.81	Shandong	100	74	54.00
Fallow	108	45	41.67	Shanghai	35	29	54.29
				Zhejiang	105	83	47.62
Total	540	269	49.81^*b*^	Total	540	269	49.81^*b*^

^*a*^ Count of EPF positive samples (containing EPF).

^*b*^ Means.

### Difference of EPF species distributed in habitats and regions

**Fig 1:**
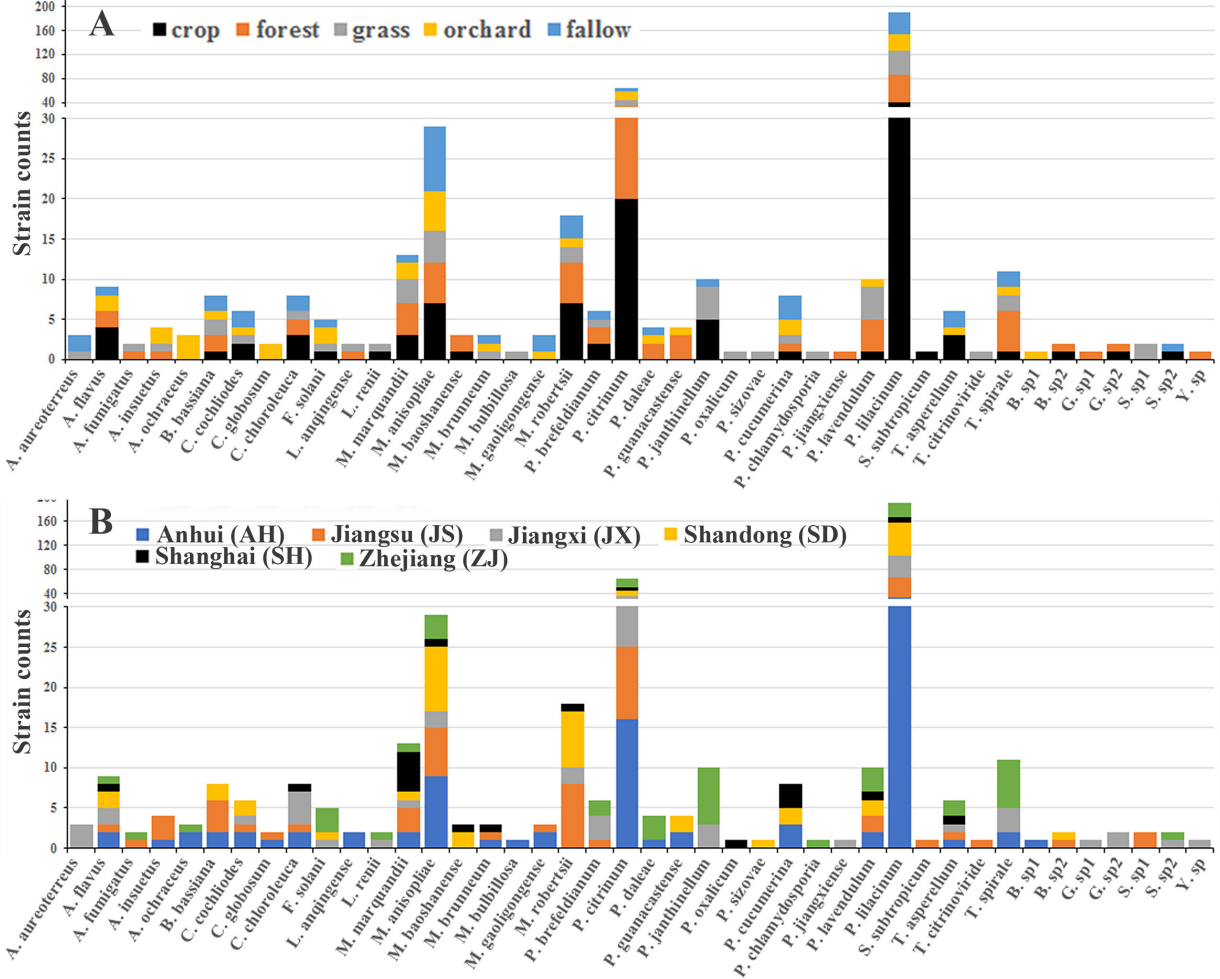
Distribution of EPF species in different habitats (**A**) and regions (**B**).

All the 42 EPF species in 18 genera belonged to the subphylum Pezizomycotina in the phylum Ascomycota, except for the genus *Gongronella* from the order Mucorales (phylum Mucoromycota). Furthermore, most taxa came from the order Hypocreales in Pezizomycotina, which included 323 strains of 24 species in 14 genera (Table 1 and Fig. 1). There were 71, 16, and 201 strains from the families Clavicipitaceae, Cordycipitaceae, and Ophiocordycipitaceae, respectively, which are generally accepted as the most common EPF.

Interestingly, *P. lilacinum* was the dominant species with 190 strains (41.76% of all EPF) (Table 1 and Fig. 1) in all habitats and regions. This species was widely distributed across five habitat types (Fig. 1A), with the highest number of strains isolated from forests (46 strains) and the lowest number from orchards (28 strains). Meanwhile, it was also distributed in different regions, especially in Shandong, where 56 strains were isolated (Fig. 1B). The phylogenetic analysis based on the sequences of ITS indicated that this species had large diversity (Fig. S3), which suggests that this species complex may contain multiple cryptic species.

Subsequently, *P. citrinum* and *M. anisopliae* were the species with the second and third highest number of strains, 65 and 29 strains, respectively. Their distribution across five different habitats also varied (Fig. 1A). The former had 8 strains (lowest) in fallow land and 20 (highest) strains in crop land, while the latter had 4 strains (lowest) in grassland and 8 (highest) strains in fallow land. Meanwhile, their distribution across six different regions varied as well (Fig. 1B). The other 39 species had 1–18 strains, and their distribution in different habitats and regions varied as well (Fig. 1).

### Diversity analysis of EPF in soils of eastern China

The analysis of the alpha and beta diversity indices was evaluated (Tables 4 to 6). Overall, the data demonstrated that the variation in the indices between the five habitat types (crop, forest, grass, orchard, and fallow) significantly exceeded those between the six regions (AH, JS, JX, SD, SH, and ZJ).

**TABLE 4:** Alpha diversity indices in different habitats and regions^*a*^

	Taxa-S	Individuals	Dominance-D	Simpson-1-D	Shannon-H	Brillouin	Menhinick	Pielou-J	Berger-Parker	Chao-1
Habitat										
Crop	22	107	0.18	0.82	2.33	1.98	2.13	0.75	0.37	40.16
Forest	23	108	0.21	0.79	2.34	1.98	2.21	0.75	0.43	28.10
Grass	26	89	0.22	0.78	2.43	1.96	2.76	0.74	0.45	49.73
Orchard	21	72	0.19	0.81	2.38	1.91	2.48	0.78	0.39	28.75
Fallow	20	79	0.22	0.78	2.27	1.85	2.25	0.76	0.46	23.46
Region										
AH	22	90	0.17	0.83	2.42	2.00	2.32	0.78	0.37	23.73
JS	21	83	0.19	0.81	2.35	1.93	2.31	0.77	0.41	42.73
JX	20	79	0.23	0.77	2.26	1.84	2.25	0.75	0.46	27.11
SD	15	97	0.33	0.67	1.78	1.51	1.52	0.66	0.56	15.74
SH	13	33	0.14	0.86	2.31	1.72	2.26	0.90	0.30	47.91
ZJ	17	73	0.15	0.85	2.35	1.96	1.99	0.83	0.32	23.90

^*a*^ Taxa-S (species richness): number of distinct species observed in the community (S). Individuals: total number of individuals in the community (N), often used to quantify community size. Dominance-D: reflects the dominance of a few species in the community, $D=\sum (ni/N{)}^{2}$, where *n_i_* is the number of individuals of the *i* species, and *N* is the total number of individuals. Simpson-1-D: probability that two randomly selected individuals belong to different species, Simpson-1-D = 1 − D. Shannon-H: based on information theory, incorporating both richness and evenness, $H^{\prime }=-\sum (P_{i}\cdot lnP_{i})$, where *P_i_* is the proportion of individuals of the *i* species. Brillouin: similar to Shannon index but applied to directly counted biological samples, HB=ln(N!)−∑ln(ni!), where *N* is the total number of individuals and *n_i_* is the count of the *i* species. Menhinick: species richness scaled by the square root of sample size, $Dmn=S/\sqrt{N}$, where *S* is the number of species, and *N* is the total number of individuals. Pielou-J: measures the uniformity of species abundance distribution, $J^{\prime }=H^{\prime }/\;lnS$, where *H′* is the Shannon index, and *S* is the number of species. Berger-Parker: reflects the dominance of the most abundant species, $d=n_{max}/N$, where *n*_max_ is the count of the most abundant species, *N* is the total number of individuals. Chao-1: estimates total species richness, including unobserved species,*S*_chao 1_= *S*_obs_+ *n*_1_(*n*_1_ − 1)/2(*n*_2_ + 1), where *S*_obs_ is the observed species richness, *n*_1_ is the number of singletons, and *n*_2_ is the number of doubletons.

**TABLE 5:** Comparison of beta diversity between habitats^*a*^

	Whittaker index						Routledge index				
	Crop	Forest	Grass	Orchard	Fallow		Crop	Forest	Grass	Orchard	Fallow
Crop	0.00	0.33	0.38	0.40	0.24	Crop	0.00	0.10	0.11	0.12	0.07
Forest	0.33	0.00	0.43	0.41	0.44	Forest	0.10	0.00	0.13	0.12	0.13
Grass	0.38	0.43	0.00	0.45	0.35	Grass	0.11	0.13	0.00	0.13	0.10
Orchard	0.40	0.41	0.45	0.00	0.27	Orchard	0.12	0.12	0.13	0.00	0.08
Fallow	0.24	0.44	0.35	0.27	0.00	Fallow	0.07	0.13	0.10	0.08	0.00

^*a*^ Whittaker index: ratio of total species richness to average community richness, $\beta_{\omega }=(S_{total}/\;\bar{s})-1$, where *S*_total_ is the total species richness across all communities, and $\bar{S}$ is the mean species richness per community. Routledge index: measures compositional dissimilarity based on species probability distributions,$\beta_{R}=2\times \left(1-\sum (P_{ij}\cdot P_{ik})\right)$, where *P_ij_* and *P_ik_* are the relative abundances of species *i* in communities *j* and *k*, respectively. Where the value is larger, the difference between the two habitats is bigger.

**TABLE 6:** Comparison of beta diversity between regions

	Whittaker index							Routledge index					
	AH	JS	JX	SD	SH	ZJ		AH	JS	JX	SD	SH	ZJ
AH	0.00	0.35	0.57	0.46	0.43	0.49	AH	0.00	0.10	0.17	0.13	0.11	0.14
JS	0.35	0.00	0.51	0.44	0.41	0.53	JS	0.10	0.00	0.15	0.13	0.11	0.16
JX	0.57	0.51	0.00	0.54	0.52	0.35	JX	0.17	0.15	0.00	0.16	0.15	0.10
SD	0.46	0.44	0.54	0.00	0.36	0.56	SD	0.13	0.13	0.16	0.00	0.11	0.17
SH	0.43	0.41	0.52	0.36	0.00	0.53	SH	0.11	0.11	0.15	0.11	0.00	0.16
ZJ	0.49	0.53	0.35	0.56	0.53	0.00	ZJ	0.14	0.16	0.10	0.17	0.16	0.00

Species richness (Taxa-S) indicated that grass habitat exhibited the highest species richness (26), the other habitats followed with slight variation (20–23). With respect to regions, the lowest richness was observed in SH (13) and SD (15) (Table 4). AH and JS regions showed moderate richness (22 and 21, respectively), while in the JX and ZJ samples, an intermediate number of taxa were recovered (Table 4).

The grass habitat had the highest Shannon index (2.43), indicating greater species diversity and evenness. SD had the lowest value (1.78), reflecting dominance by a few species in this region. AH (2.42) and ZJ (2.35) showed relatively high diversity, while SD’s low score aligns with its low species richness (Table 4).

Furthermore, dominance and evenness can be estimated by the indices of Dominance-D and Pielou’s evenness index (J). Dominance-D was highest in grassland (0.22) and SD (0.33), indicating stronger dominance by a few species. Conversely, SH (0.14) and ZJ (0.15) had lower dominance, suggesting more even distributions. Pielou’s evenness index (J) was highest in SH (0.90) and ZJ (0.83), indicating uniform species abundance, while SD (0.66) had the least evenness (Table 4).

Based on the Chao-1 index, grassland and SH had the highest estimated total species richness (49.73 and 47.91, respectively), implying potentially undiscovered species. SD’s low Chao-1 index (15.74) suggested limited hidden diversity.

In summary, the alpha diversity analysis discovered that grassland habitat and the AH region supported higher EPF biodiversity, while SD and SH may face ecological constraints.

On the other hand, the differences between habitats were exhibited through beta diversity analysis (Table 5). The Whittaker indices showed that the highest dissimilarity occurred between orchard-grass (0.45) and forest-fallow (0.44), indicating significant species turnover. Crop-fallow (0.24) showed the least dissimilarity. Routledge indices values mirrored Whittaker trends but at a smaller scale (e.g., orchard-grass: 0.13) (Table 5). This suggests that while species composition differs, abundance distributions were less divergent.

Whittaker indices showed that JX exhibited the highest dissimilarity with AH (0.57) and SD (0.54), highlighting distinct species pools. ZJ-JX (0.35) was the most similar pair. Routledge indices indicated that JX-AH (0.17) and ZJ-SD (0.17) had the highest values, emphasizing pronounced compositional differences (Table 6).

**Fig 2:**
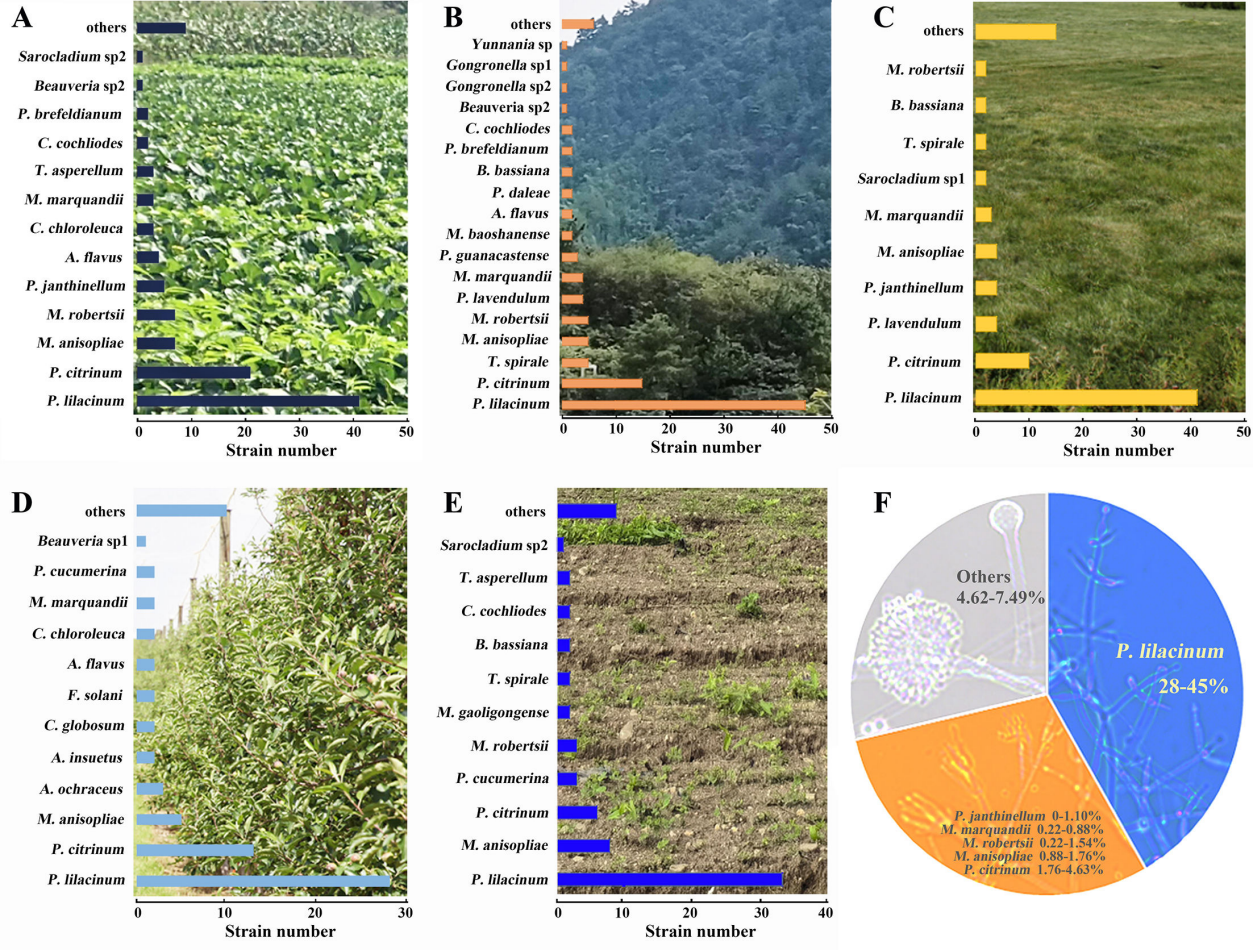
Composition and structure of entomogenous fungal communities in different habitats. (**A–E**) Crop/forest/grass/orchard/fallow lands. (**F**) The composition of entomopathogenic fungal communities across the five habitats.

Finally, analysis of community structure revealed that *P. lilacinus* was the most abundant species across all habitats, comprising 28%–45% of all species (Fig. 2). It was followed by *P. citrinum*, which accounted for 1.76%–4.63% of isolates and ranked either second (Fig. 2A through D and F) or third (Fig. 2E). *M. anisopliae* with 0.88%–1.76% ranked in the second (Fig. 2E and F) and third-fifth order (Fig. 2A through D). Notably, *M. marquandii* appeared among the top 10 in all habitats except fallow land (Fig. 2); *P. janthinellum* ranks 4th to 5th in crop and grassland habitats (Fig. 2A and C), but fell below 10th place—or was even absent—in other habitats (Fig. 2B, D, and E); while *T. spirale* ranked 3rd, 7th, and 8th, respectively, in forest, grass, and fallow habitats (Fig. 2B and C).

In summary, the diversity analysis results indicated that the natural habitats (forest and grassland) exhibited higher EPF diversity than the agricultural habitats (cropland, orchard, and fallow lands), which implies that natural habitats provide a richer range of ecological niches and more stable environmental conditions for EPF. However, the community structure of EPF across different habitats showed certain similarities. Specifically, *P. lilacinum* was identified as the dominant species in all habitats, while *P. citrinum* and *M. anisopliae* were the minor dominant species in all habitats. In contrast, *M. robertsii*, *P. janthinellum*, *Mar. marquandii*, and *T. spirale* exhibited variable dominance depending on the habitat.

## DISCUSSION

The present study reveals distinct patterns of EPF diversity across five habitat types: crop, fallow, forest, grass, and orchard lands. Overall, EPF diversity is slightly higher in natural habitats (forest and grasslands) than in agricultural lands (cropland, orchard, and fallow), particularly in terms of species richness. The results indicate that agricultural practices had a lower EPF diversity compared to natural habitats, largely due to anthropogenic disturbances—such as monocropping, fertilization, pesticide use, and irrigation—that significantly alter EPF distribution patterns. Notably, natural habitats (grass and forest lands) support higher EPF species diversity and evenness than agricultural ones. However, cropland and forest habitats showed similar values for the number of individuals (107 and 108, respectively) and Taxa-S (22 and 23). This similarity may be attributed to the high plant biomass in both habitats, which supports larger insect populations and thus promotes EPF occurrence. For instance, common EPF species such as *M. anisopliae*, *M. robertsii*, and *P. citrinum* were more frequently isolated from crop land than from forest or grasslands. This may result from the simplified plant structure and lower insect diversity in agricultural systems, where certain pest species proliferate and facilitate the dominance of EPF. In contrast, orchard and fallow land exhibited lower alpha diversity, with Taxa-S values of 21 and individual counts of 72 and 79, respectively. This reduction is likely due to the absence of host crops in fallow land, sparse vegetation, and limited insect activity, all of which constrain EPF establishment and abundance. Conversely, forest habitats displayed the highest EPF diversity, an outcome likely associated with greater biodiversity, ecosystem stability, and minimal anthropogenic interference (41).

In this study, *P. lilacinum* was identified as the dominant species across nearly all surveyed habitats and regions, a finding consistent with our previous reports (8, 13). In contrast, the closely related species, such as *P. lavendulum* and *P. jiangxiense*, exhibited considerably more restricted distributions. The broad prevalence of *P. lilacinum* is likely attributable to its strong capacity to adapt to diverse environmental conditions, including variations in temperature, humidity, and soil pH. This species is widely applied as a commercial nematicide and has also demonstrated insecticidal activity against a range of insect pests. Furthermore, our results reveal considerable genetic differentiation within *P. lilacinum*, suggesting that it may represent a species complex comprising multiple cryptic species.

It is estimated that over 1,000 species of EPF have been identified, with new species continually being discovered. The largest group of EPF belongs to the phylum Ascomycota, particularly the order Hypocreales, although representatives are also found in Entomophthoromycota, Mucoromycota, Microsporidia, and other phyla (1). However, in practical control of pest insects and nematodes, currently only ~30 EPF species are applied in the world (2). These primarily originate from families within Hypocreales, such as Cordycipitaceae and Clavicipitaceae, mainly due to their high entomopathogenicity and their demonstrated effective insecticidal properties in fields. This suggests that broader application of EPF requires further efforts. Interestingly, a considerable proportion of EPF species are opportunistic insect pathogens, primarily originating from non-hypocrealean fungi. In the laboratory, opportunistic EPF exhibit some pathogenic activity against insects, but their insecticidal efficacy is often low in fields, consequently leading them to be easily overlooked. However, the functional role of these fungi in natural ecosystems remains largely unknown. It is plausible that they contribute to the natural regulation of pest populations in soil environments. For instance, newly hatched or physiologically compromised insects—such as malnourished individuals—may be susceptible to infection by these fungi when in contact with soil. Moreover, some opportunistic EPF may indirectly affect insect populations through plant-mediated mechanisms, including endophytic colonization or rhizosphere interactions. Such hypotheses, however, require further experimental validation.

In this study, we isolated and identified 455 strains of EPF, classified into 42 species and 18 genera, many of which were the same as those found in previous studies conducted in Central, South, and Southwest China (8, 12, 13). In practice, we identified several factors that influence the EPF isolation. Firstly, isolating fungi from excessively moist soils is challenging, because high humidity severely affects fungal survival in the soil (42). Secondly, the storage conditions of soil samples are critical. Typically, storing soil samples at 0°C for 3–6 months does not affect fungal isolation from the soil; however, prolonged storage leads to a rapid decrease in the number of fungi present in the samples. Furthermore, repetitive operations also affect the isolation results of fungal species in each sample, and repeating the process twice can significantly increase the number of isolated fungal species. Variations in collection sites and sampling times may also contribute to discrepancies in the results (43). This study has effectively controlled the aforementioned factors; therefore, the research findings presented are credible and reliable. Undoubtedly, our findings provide the first comprehensive data set on the species and strains of soil-borne EPF from these regions.

This study suggested seven new species, four of which (*B*. sp1, *B*. sp2, *S*. sp1, and *S*. sp2) are from the order Hypocreales, while the other three species (*G*. sp1, *G*. sp2, and *Yun*. sp.) (in recent, they were published respectively with the name as *G. shangraoensis*, *G. yichunensis*, and *Y. jiujiangensis*) (10) are non-Hypocrealean. The two new *Beauveria* species exhibited high insecticidal activity against the ACB (*O. furnacalis*) comparable to that of *B. bassiana* strains, suggesting potential biocontrol applications, though further research is warranted. However, the two *Beauveria* species have to be described in the future. The other three new species—*G*. sp1, *G*. sp2, and *Y*. sp.—demonstrated limited insecticidal activity. However, these species represent the first reported EPF within their respective genera. Notably, *Gongronella* (order Mucorales) is particularly significant, as fungal insecticidal activity within this order is rarely documented. Furthermore, *Sarocladium* spp. were often reported as plant pathogens and cases of human infection, but there were no reports about these fungi as EPF. The genus *Yunnania* was established in 1998; there are three species to date, and no insecticidal strains were found. Therefore, our findings hold scientific value, as they expand the known diversity of EPF and highlight understudied taxonomic groups. The applied potential of these species, particularly in biocontrol or ecological studies, merits further investigation.

Of course, this study also has several limitations. For instance, soil sampling was confined to the period of June and July. Given that organisms, including EPF, exhibit seasonal fluctuations, this narrow sampling window undoubtedly constrains the comprehensiveness of our findings. Furthermore, the bioactivity assays for the putative new fungal species were conducted solely on a single pest, the ACB (*O. furnacalis*). The activity of EPF can vary significantly across different fungal species and strains, as well as their target organisms; thus, the narrow scope of this bioassay limits the generalizability of the results.

In conclusion, we identified 455 strains of EPF in 42 species and 18 genera isolated from 540 soil samples from 108 locations with 5 types of habitats. The habitat types seriously influence the EPF distribution, but the diversity of EPF across different geographic regions (provinces/municipalities) exhibited little variation. Overall, the EPF diversity in the natural habitats (grass and forest lands) has higher species richness compared to the agricultural habitat (crops, orchards, and fallow lands). Whereas, the findings demonstrate the similarity of the EPF community structures among different habitats or regions. The species, *P. lilacinum*, was dominant in various habitats and regions, which may be due to underlying significant genetic differentiation. Additionally, many new EPF species and strains were found and showed potential as fungal biocontrol agents. These findings provide new insights to understand the fungal diversity. It can promote the research and application of EPF.

## MATERIALS AND METHODS

### Collection of soil samples

Soil samples were collected in June and July 2023. The sampling sites with five habitat types namely crop (A), forest (B), grass (C), orchard (D), and fallow lands (E) ranged across five regions (province and municipality), namely, Anhui (AH), Jiangsu (JS), Jiangxi (JX), Shandong (SD), Zhejiang (ZJ), and Shanghai (SH) in eastern China (Fig. S1). The longitude and latitude of each site were recorded by ICEGPS 100C (Shenzhen, China). When sampling, five points in each habitat were randomly selected. At each site, we established five sampling points spaced at least 50 m apart to ensure spatial independence. The surface layer was removed, and the soil at ~20 cm depth was collected. All of the soils (~200 g) from each habitat were packaged individually in re-sealable plastic bags. Every sample was named based on the “region + site number + habitat type” (e.g., AH01A, indicating the sample collected from Anhui 01 site in cropland habitat). The samples were preserved at 4°C for further use within 1 month.

### Isolation and purification of fungi from soil

Collected soil samples were sieved through a 0.45 mm mesh to remove coarse particles. Two aliquots of 10 g each were subsequently mixed with 100 mL of 0.1% Tween-80 solution, vortexed thoroughly, and allowed to stand for 10 min to prepare soil suspensions. From each suspension, 100 μL was inoculated onto a selective plate (comprising 200 g potato extract, 20 g glucose, 20 g agar, 50 mg chloramphenicol, 50 mg streptomycin, 50 mg Rose Bengal, and 1 L distilled water), and evenly spread across the surface. Each treatment was performed in triplicate. The plates (six plates in each sample) were incubated at 26 ± 1°C in a constant temperature chamber. Upon the appearance of single colonies on the selective plates, the colonies were transferred to potato dextrose agar (PDA) plates using an inoculation loop for pure culture isolation. This process was repeated until pure fungal strains were obtained. Purified strains were then suspended in 20% glycerol solution and stored at −80°C (8).

### Morphological observation of fungal strains

The previous methods were used (24). In brief, purified fungal strains were inoculated onto PDA plates at 26 ± 1°C for 5–7 days. Mycelial and sporulation morphology were observed using light microscopy with a digital camera (MC-D500U, Phoenix, Jiangxi, China), and photographic documentation of colony morphology on both the obverse and reverse sides of PDA plates was performed.

### DNA extraction, PCR amplification, and sequencing

Fungal genomic DNA was extracted from colonies grown on a PDA plate for a week, using a fungal DNA extraction kit (Omega, D3195-02) and following the protocol provided by the manufacturer. The genomic loci, ITS and LSU were amplified by PCR with the primer pairs, ITS1/ITS4 and LR5/LR0R. The PCR products were validated by 1% agarose gel electrophoresis to display a clear single band, and then they were sequenced by the Sanger method (performed by Zhejiang Youkang Biotechnology Co., Ltd., Hangzhou, China).

### Molecular phylogenetic analysis

DNA sequences were uploaded to the GenBank nucleotide database at the National Center for Biotechnology Information, and the GenBank accession numbers were recorded. All obtained sequences were compared with sequences available in the GenBank database using Basic Local Alignment Search Tool. Sequence alignment was performed using the MAFFT version 7 web server (https://mafft.cbrc.jp/alignment/server/), employing the iterative optimization strategy (FFT-NS-i) for multiple sequence alignment. Subsequently, single-loci ITS/LSU phylogenetic trees were constructed using the maximum likelihood and neighbor-joining methods, implemented in MEGA 7 software (44).

### Bioactivity assays of putative new species

The insect, ACB, *O. furnacalis*, was used to test the insecticidal bioactivity of fungal strains. The ACB population had been reared for five generations with an artificial diet (43 g cornmeal, 43 g soybean flour, 26 g yeast extract, 26 g glucose, 1.5 g multivitamin complex, 5.7 g agar, and 1.5 g sorbic acid). Bioassays were conducted following the China Agricultural Industry Standard NY/T 1154.6-2006 for insecticidal bioassays in the lab, with minor modifications (24). Conidia of the tested strains were suspended in a 0.05% Tween-80 solution to a stock of 1 × 10^8^ spores/mL. The third-instar larvae of *O. furnacalis* were immersed in conidia suspension for 10 s. After dryness, the larvae were transferred to individual dishes and incubated at 25 ± 1°C. Larvae were fed with diet, which was changed every 2 days. Mortalities were recorded every day, and the dead larvae were transferred to Petri dishes for mycelial growth. Subsequently, the fungi emerging from the cadavers were re-isolated onto PDA plates and identified morphologically to confirm pathogenicity in accordance with Koch’s postulates. Control groups were treated with a 0.05% Tween-80 solution. There were three repeats with 10 larvae in each treatment. The same test was replicated twice. An ANOVA was performed using SPSS version 26.0 (IBM, USA) to test the significance. Data were compared with DMRT. Statistical significance was considered at *P* < 0.05.

### Diversity analysis

Alpha and beta diversity indices of EPF in different habitats and regions were comprehensively analyzed using PAST software, providing insights into species richness and community composition across different habitats and geographic regions. Alpha diversity refers to species diversity within a single biological community or habitat. It is commonly used to measure species richness and evenness in a specific location or ecosystem, which include Taxa-S (species richness), individuals (total number of individuals in the community), Dominance-D, Simpson-1-D, Shannon-H, Brillouin, Menhinick, Pielou-J, Berger-Parker, and Chao-1, etc. Beta diversity quantifies the variety of species composition between distinct communities or habitats, mainly including the Whittaker and Routledge indices.

## Data Availability

The newly generated DNA sequences belong to the putative new species with access numbers (Table S1) are deposited in GenBank.

## AFTER EPUB

*[This article was published on 13 February 2026 with an error in the text. The error was corrected in the current version, posted on 24 February 2026.]*

## SUPPLEMENTAL MATERIAL

Figure S1 spectrum.02904-25-s0001.pdf Sampling map.

Figure S2 spectrum.02904-25-s0002.pdf Morphology of fungal species.

Figure S3 spectrum.02904-25-s0003.pdf Phylogenetic trees.

TABLE S1 spectrum.02904-25-s0004.pdf Detailed strain information.

ASM does not own the copyrights to Supplemental Material that may be linked to, or accessed through, an article. The authors have granted ASM a non-exclusive, world-wide license to publish the Supplemental Material files. Please contact the corresponding author directly for reuse.
