# Supplementary figures and images for: Biodiversity of entomopathogenic fungi in soils of eastern China
Source: Microbiol Spectr. 2026 Feb 13;14(4):e02904-25. doi: 10.1128/spectrum.02904-25 (PMC13055385; doi:10.1128/spectrum.02904-25)

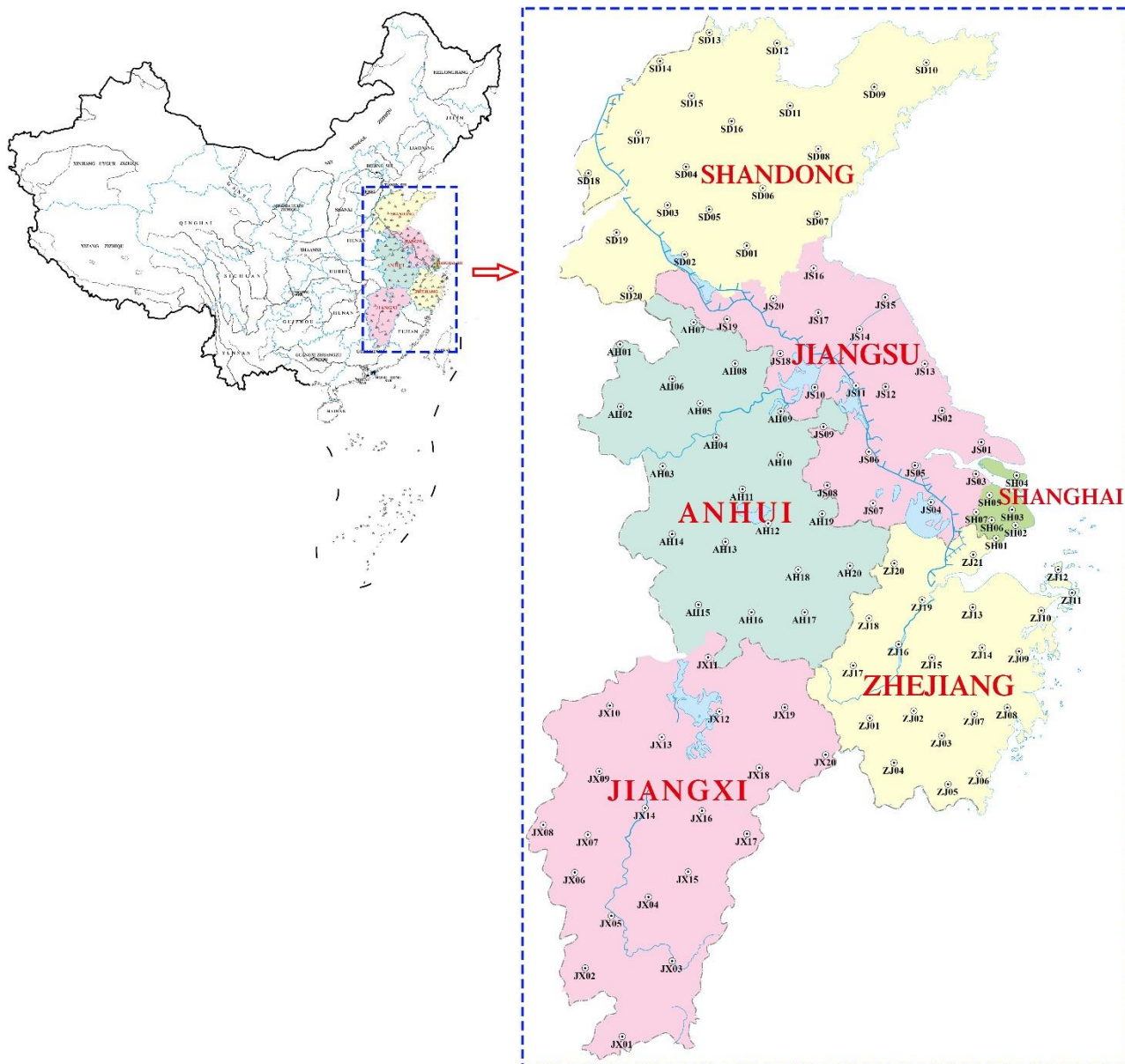

Figure S1 Soil Sample Collection Map

Supplement: Figure S1 — Sampling map. [file spectrum.02904-25-s0001.pdf]
