# Supplementary material for: Biodiversity of entomopathogenic fungi in soils of eastern China
Source: Microbiol Spectr. 2026 Feb 13;14(4):e02904-25. doi: 10.1128/spectrum.02904-25 (PMC13055385; doi:10.1128/spectrum.02904-25)
Supplement: Figure S2 — Morphology of fungal species. [file spectrum.02904-25-s0002.pdf]

Figure S2 Morphological characterization of the fungi species (In each picture: Top - colony surface on PDA plate; Middle - colony reverse on PDA plate; Bottom - microscopic features of sporulation structures.)

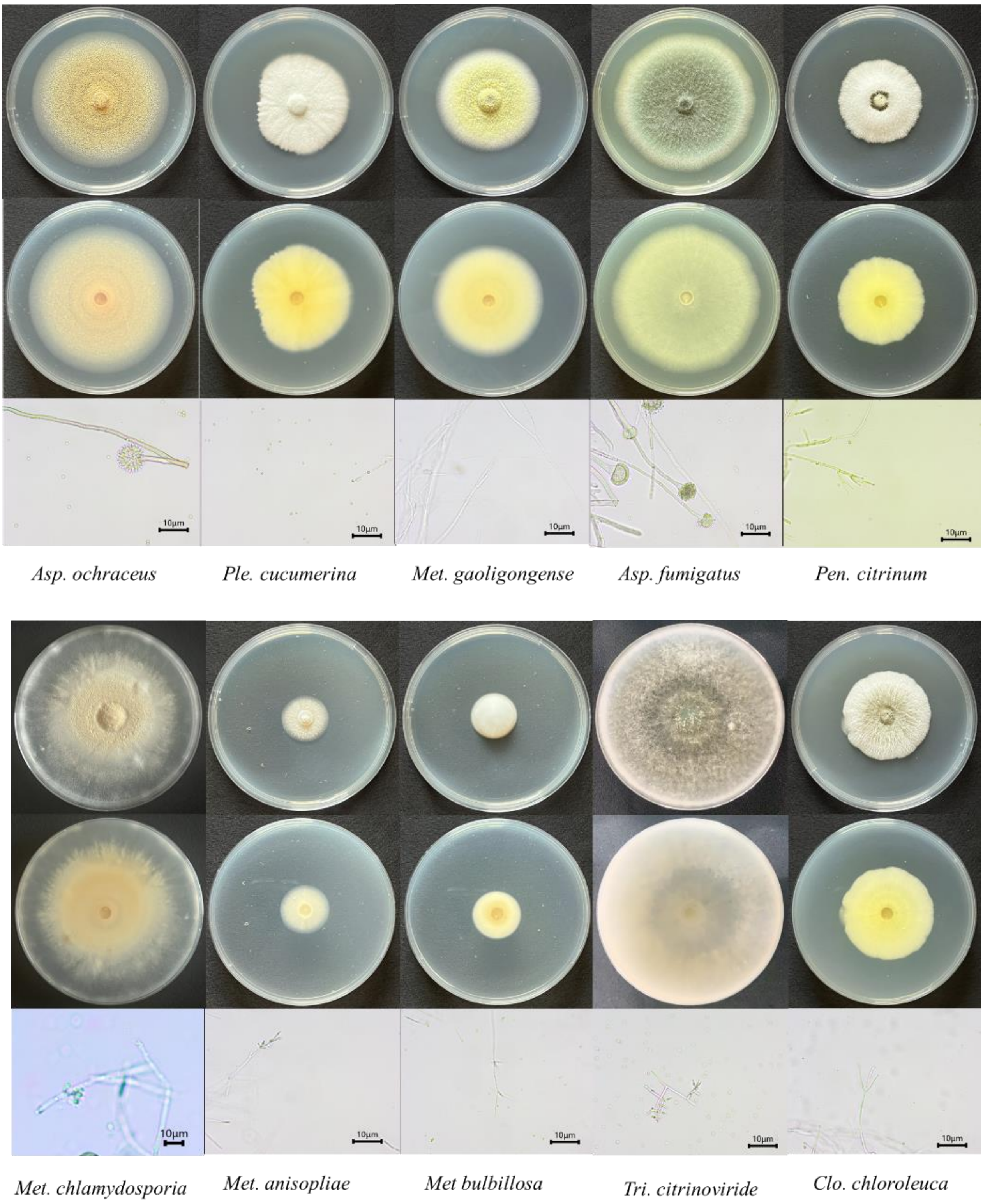

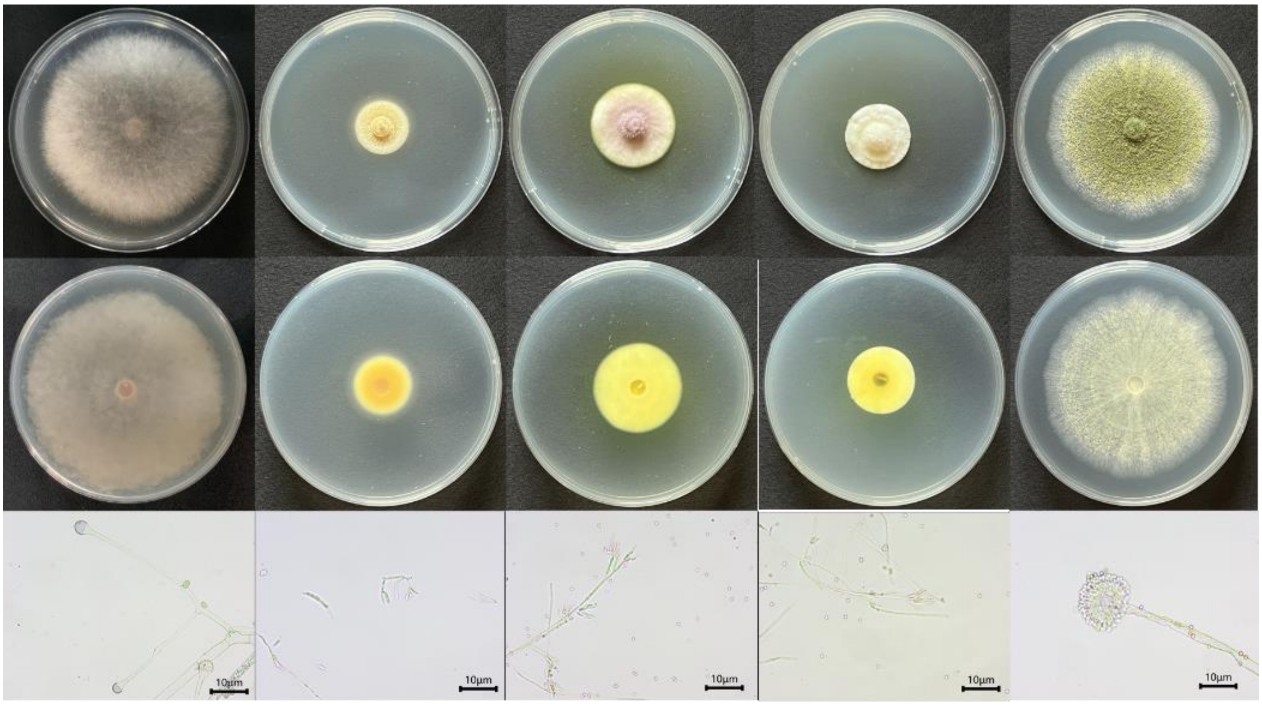

Cha. cochliodes *Met. brunneum* *Mar. marquandii* *Pen. daleae* *Asp. flavus*

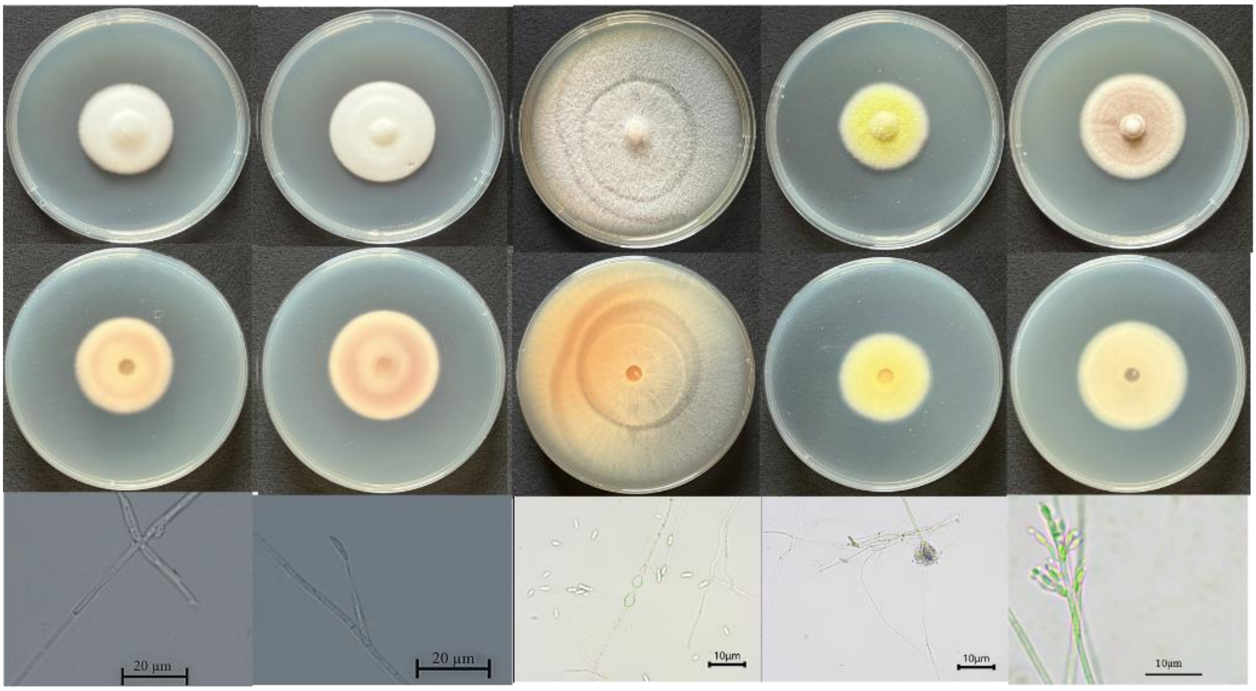

Lec. renii *Lec. anqingense* *Fus. solani* *Asp. insuetus* *Pur. jiangxiense*

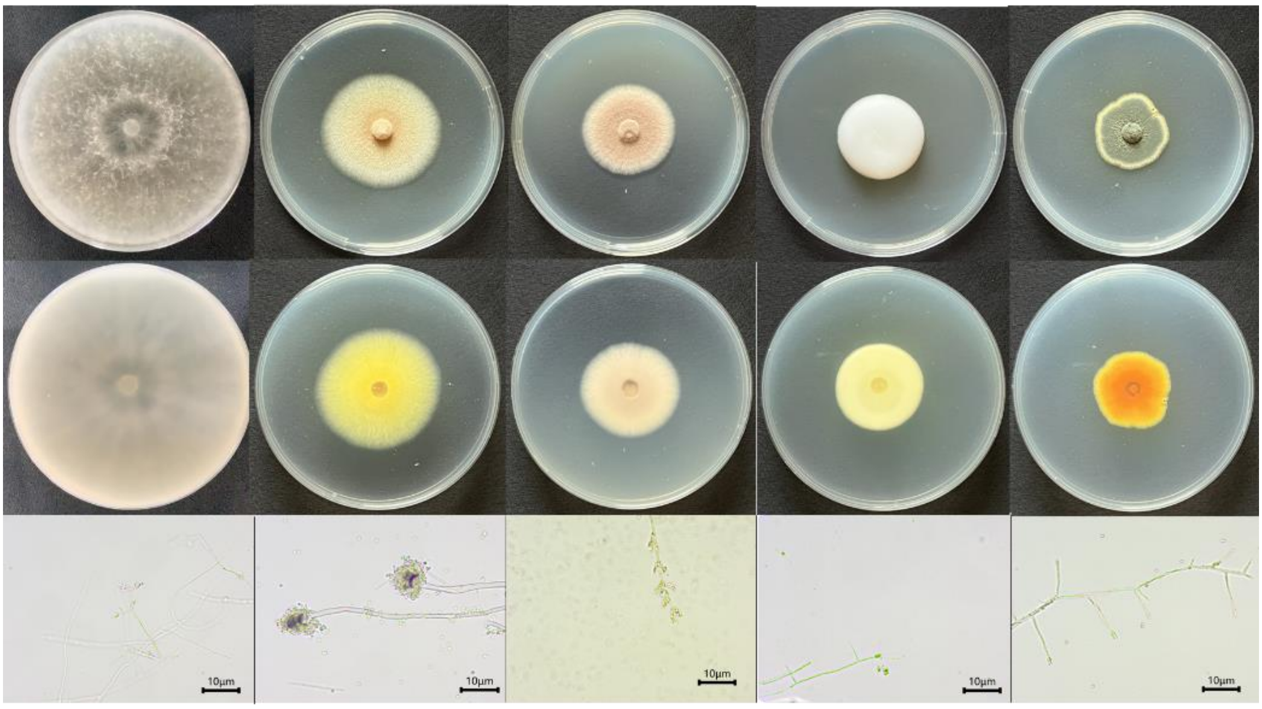

Tri. asperellum *Asp. aureoterreus* *Pur. lilacinum* *Sim. subtropicum* *Pen. guanacastense*

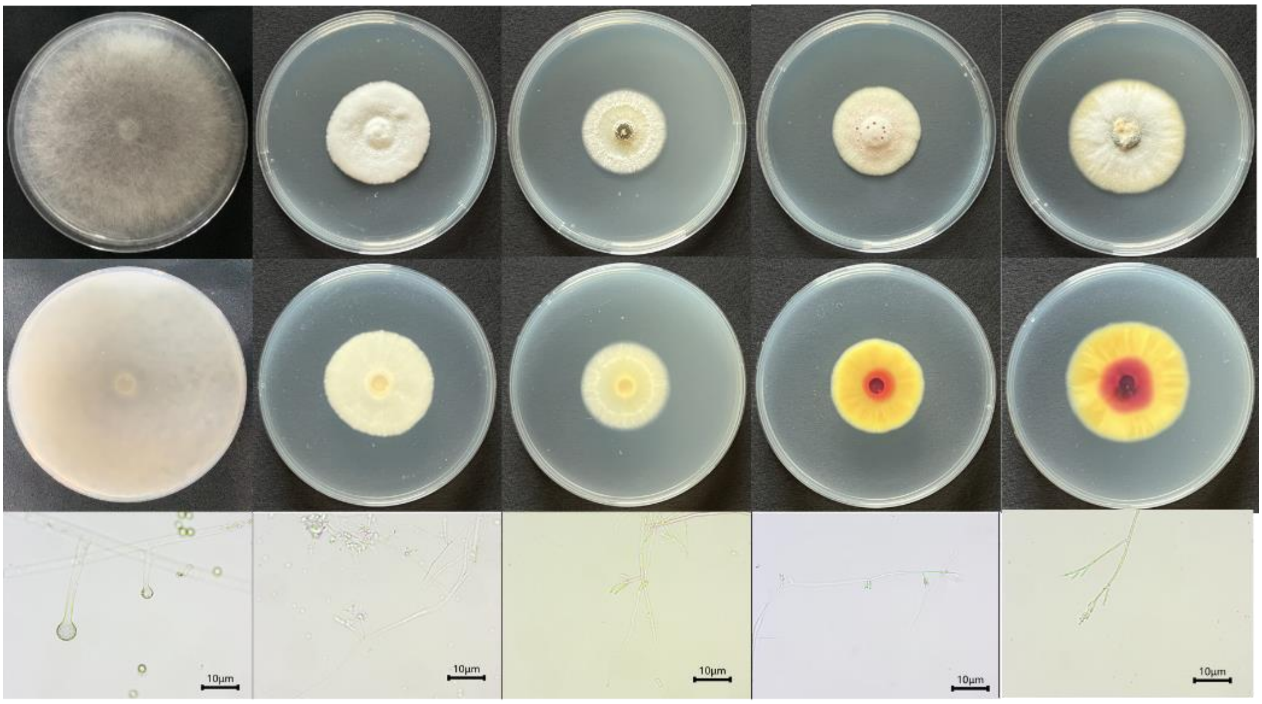

Cha. globosum *Bea. bassiana* *Met. baoshanense* *Pen. oxalicum* *Pen. janthinellum*

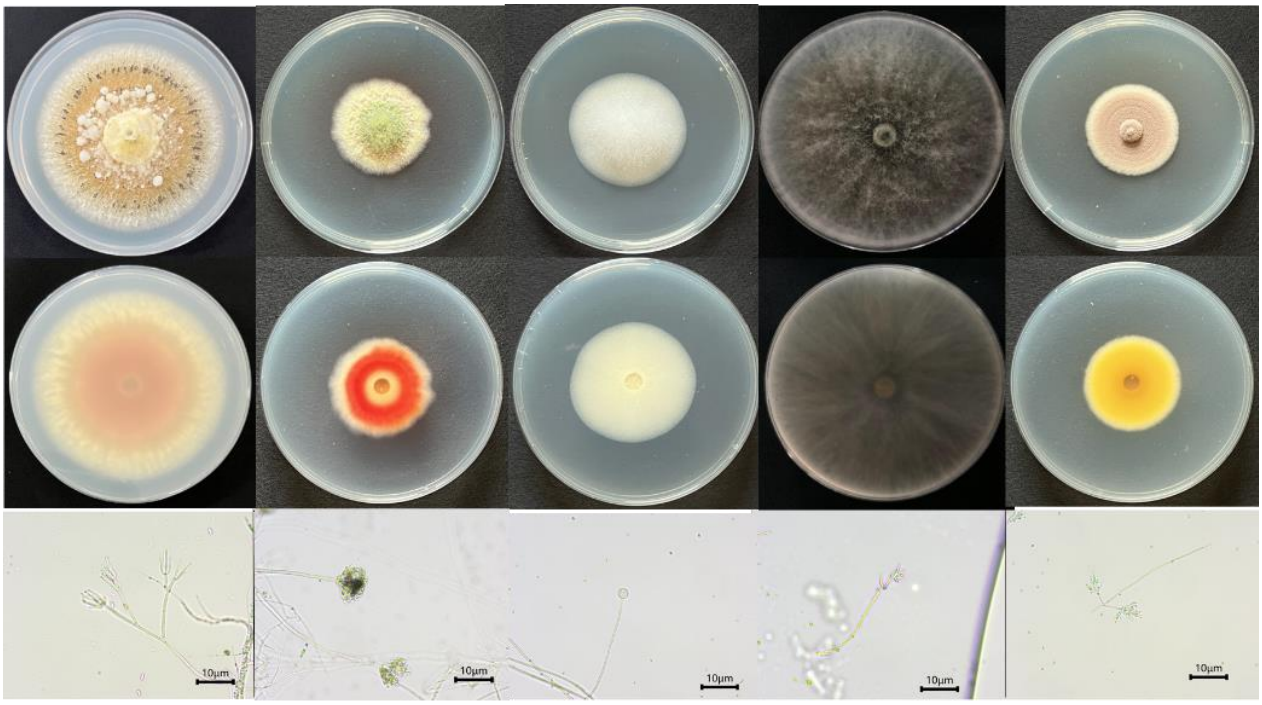

Met. robertsii *Pen. brefeldianum* *Tri. spirale* *Pen. sizovae* *Pur. lavendulum*
