# Supplementary material for: Biodiversity of entomopathogenic fungi in soils of eastern China
Source: Microbiol Spectr. 2026 Feb 13;14(4):e02904-25. doi: 10.1128/spectrum.02904-25 (PMC13055385; doi:10.1128/spectrum.02904-25)
Supplement: Figure S3 — Phylogenetic trees. [file spectrum.02904-25-s0003.pdf]

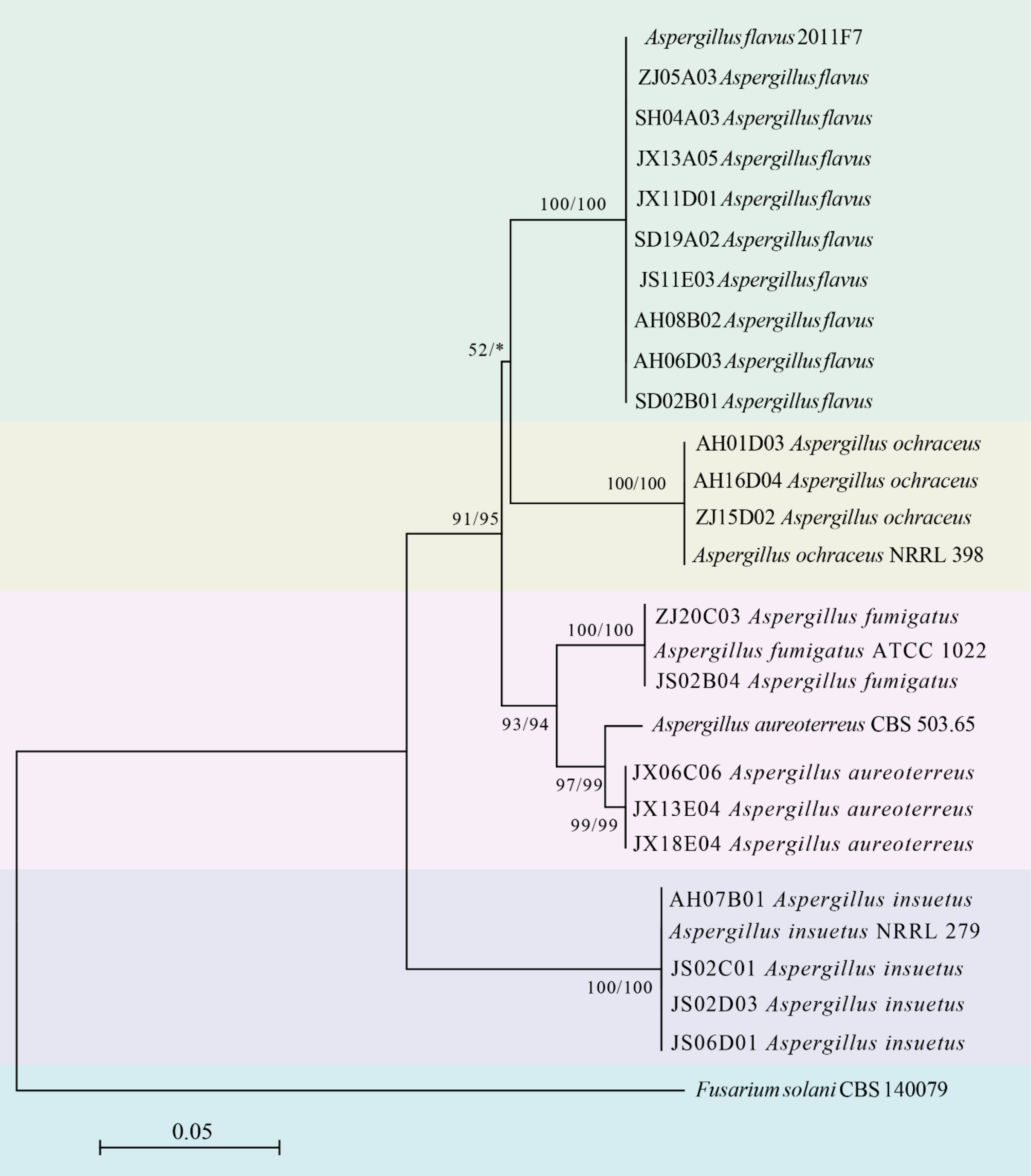

Figure S3.0 Phylogenetic tree of *Aspergillus* based on ITS gene

Note: Bootstrap support values for Neighbor-Joining (NJ) and Maximum Likelihood (ML) analyses are displayed on the branches in the order NJ/ML. NJ < 50 and ML < 50 are marked as "*". The same as below.

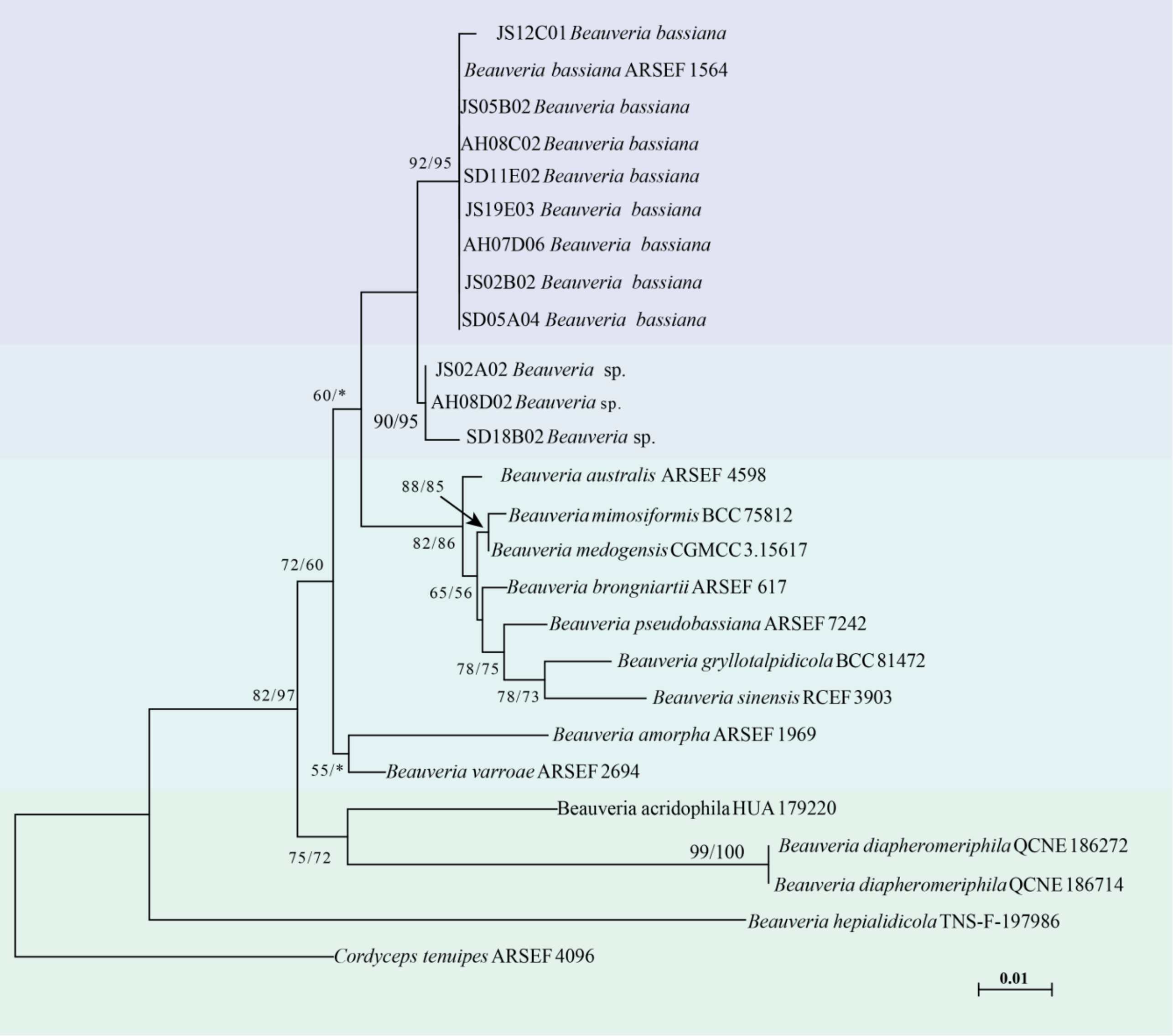

Figure S3.1 Phylogenetic tree of *Beauveria* based on ITS gene

Note: Bootstrap support values for Neighbor-Joining (NJ) and Maximum Likelihood (ML) analyses are displayed on the branches in the order NJ/ML. NJ < 50 and ML < 50 are marked as "*". The same as below.

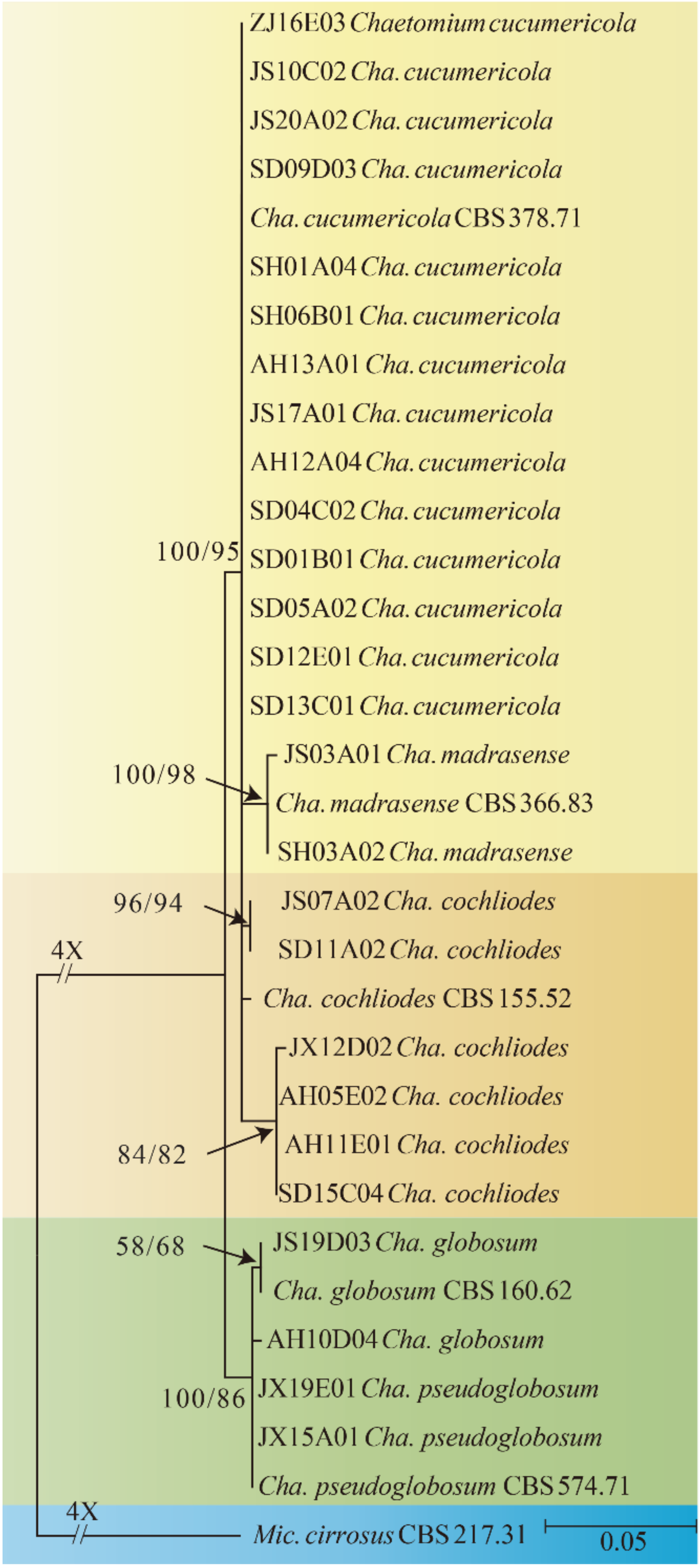

Figure S3.2 Phylogenetic tree of *Chaetomium* based on ITS gene

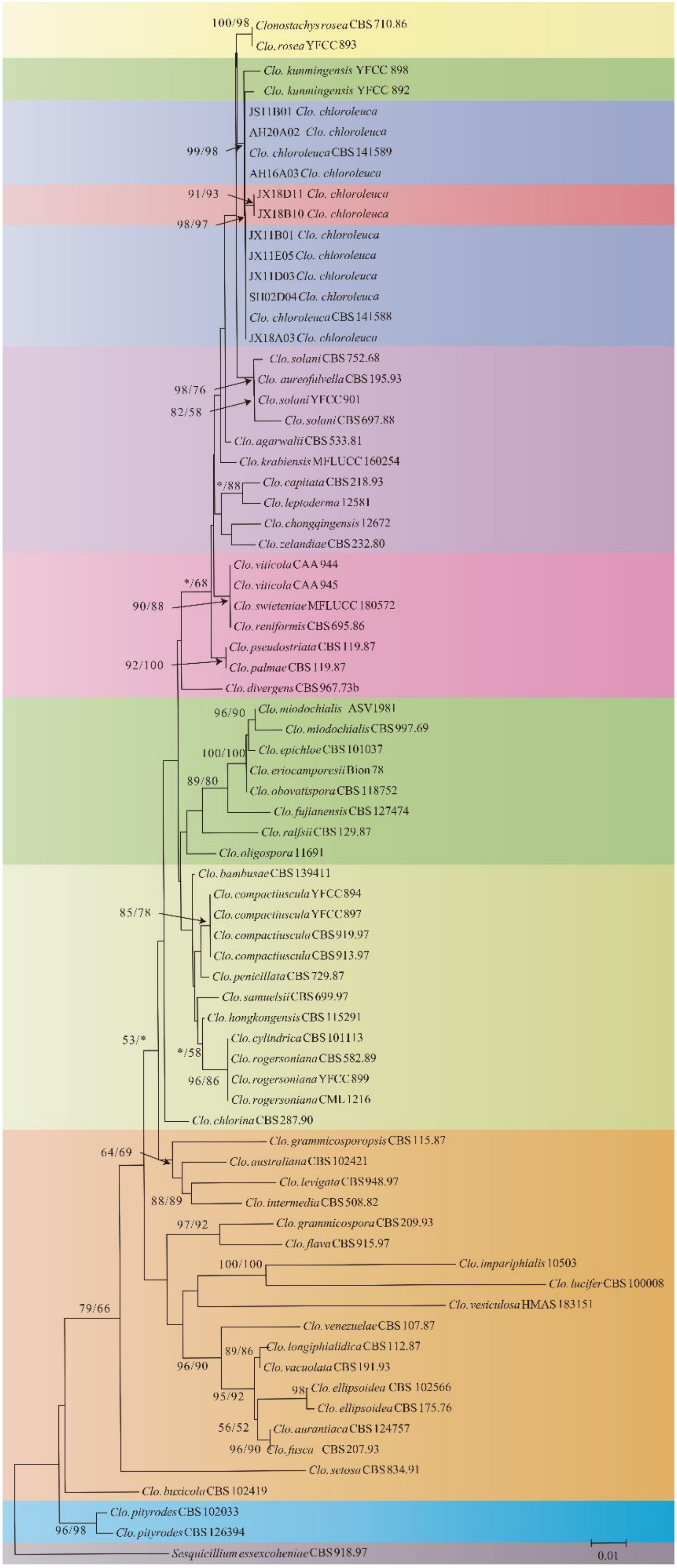

Figure S3.3 Phylogenetic tree of *Clonostachys* based on ITS gene

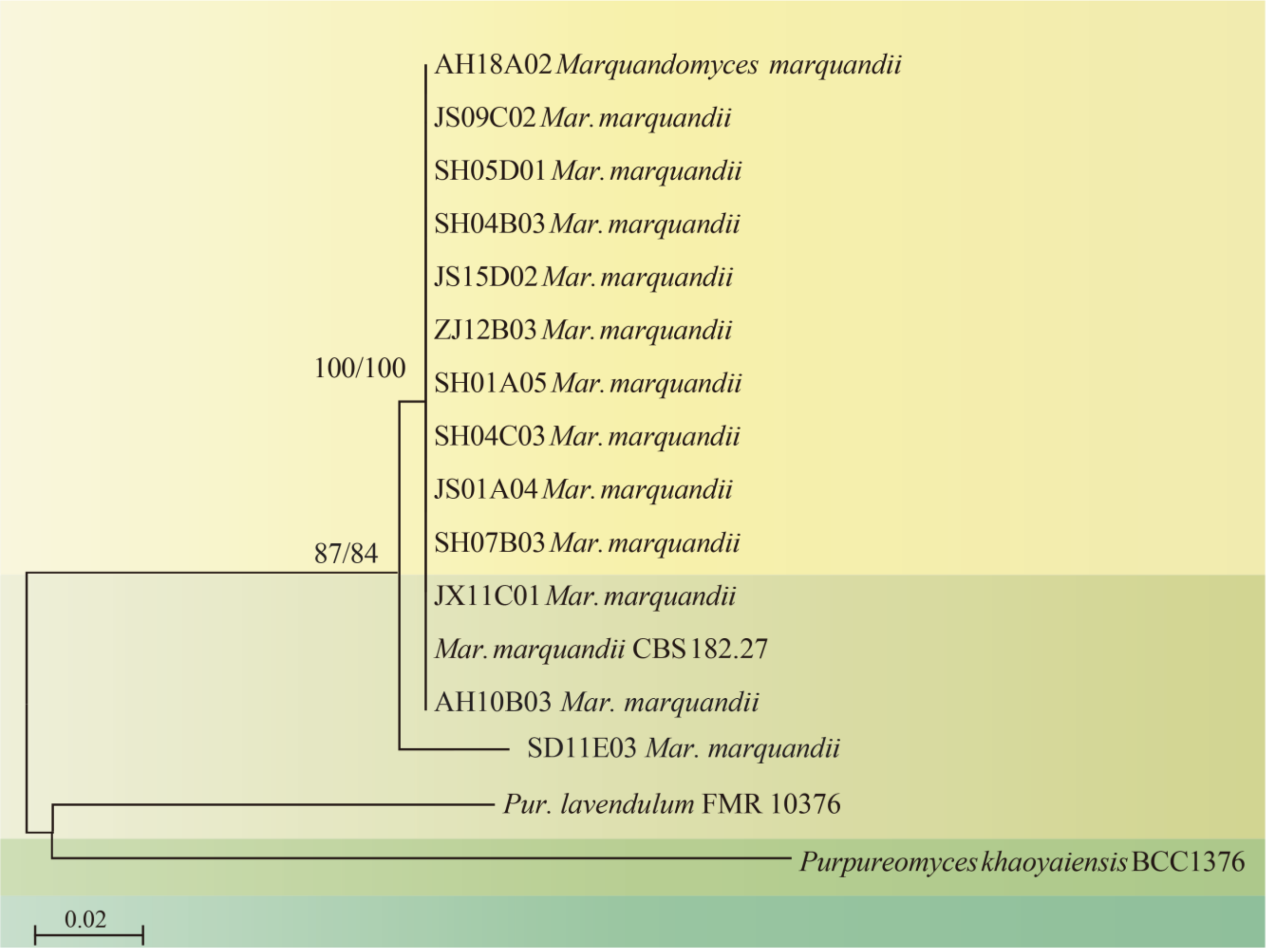

Figure S3.4 Phylogenetic tree of *Marquandomyces* based on ITS gene

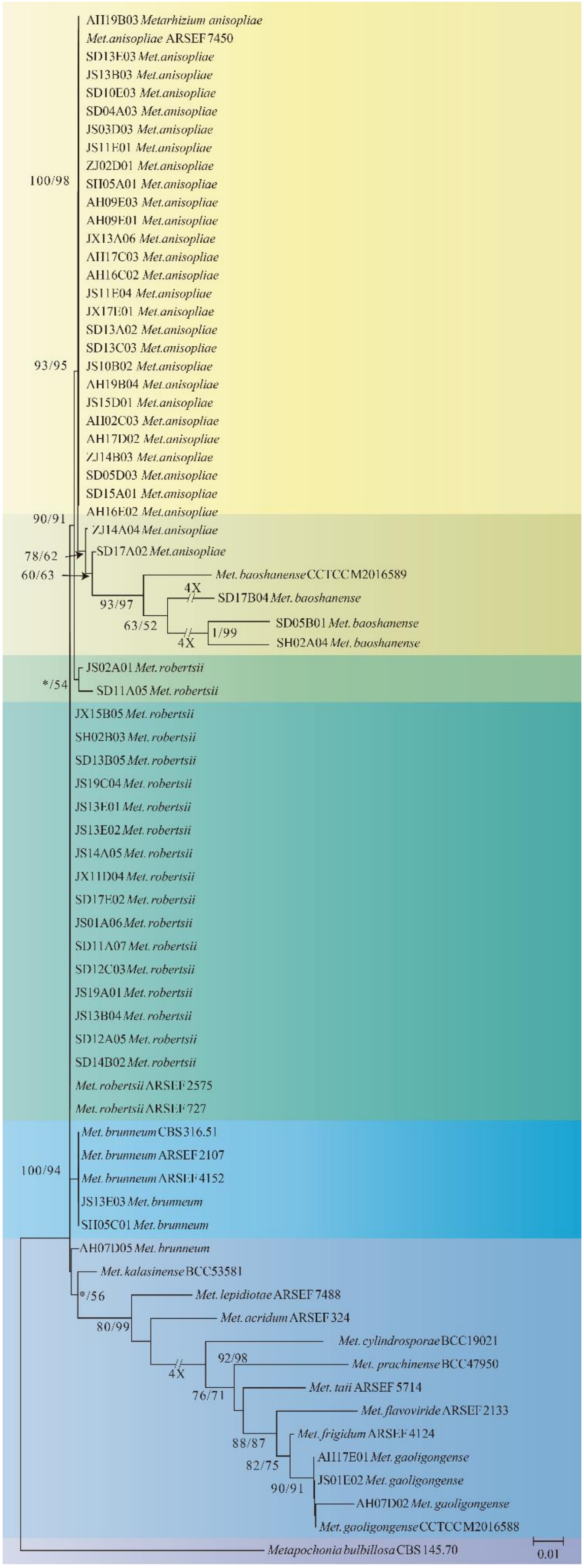

Figure S3.5 Phylogenetic tree of *Metarhizium* based on ITS gene

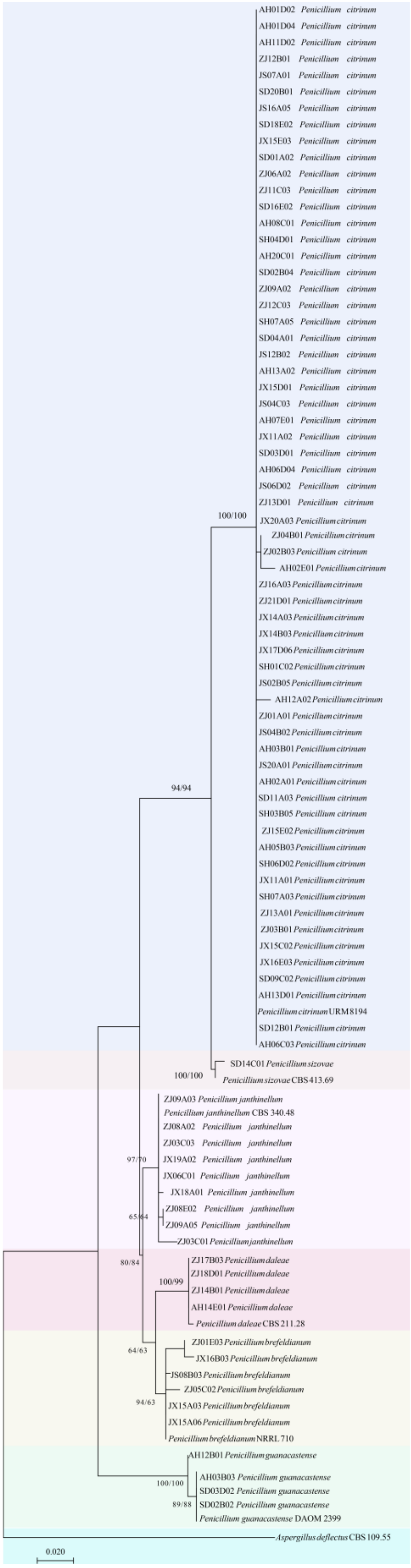

Figure S3.6 Phylogenetic tree of *Penicillium* based on ITS gene

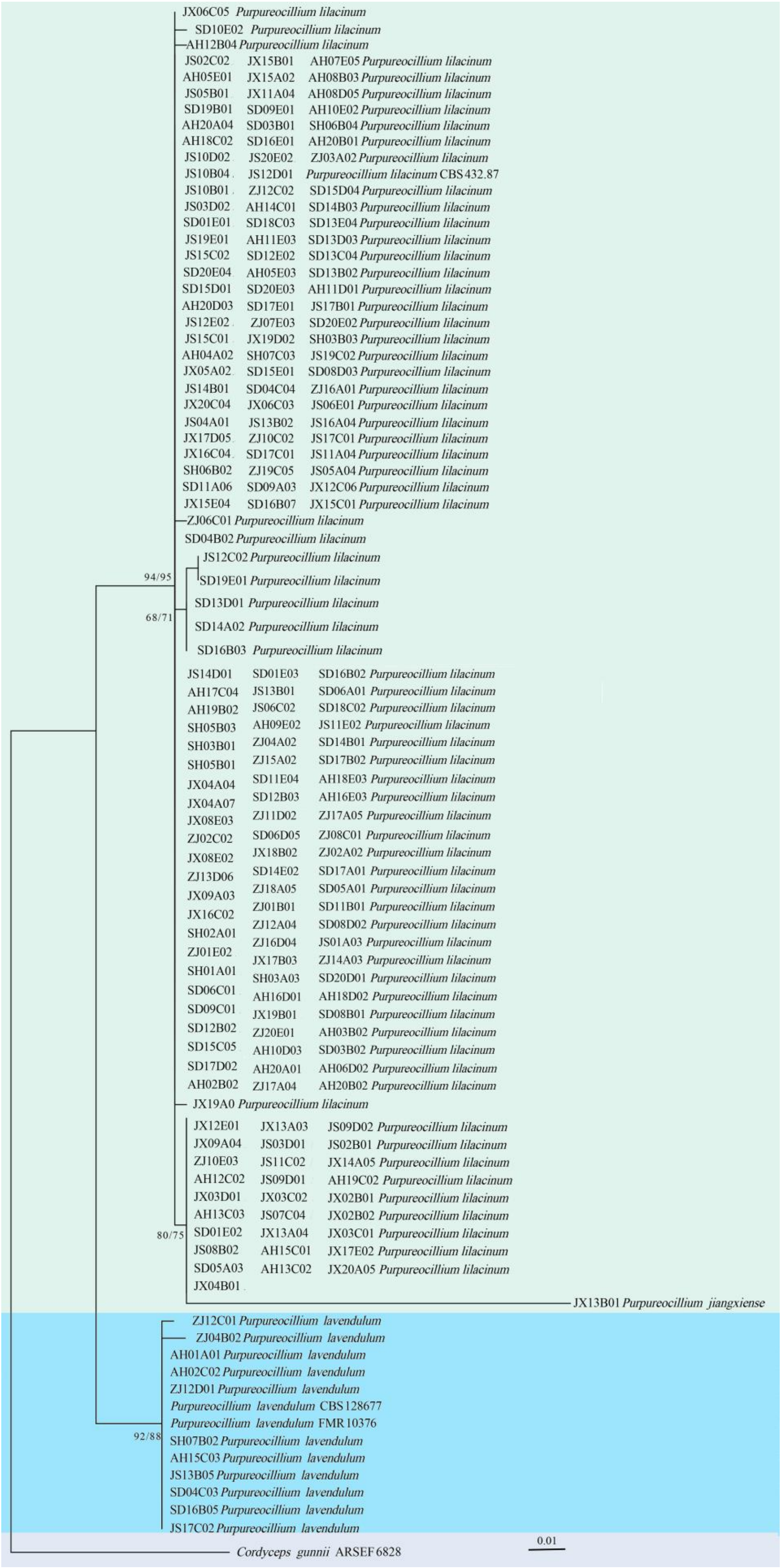

Figure S3.7 Phylogenetic tree of *Purpureocillium* based on ITS gene

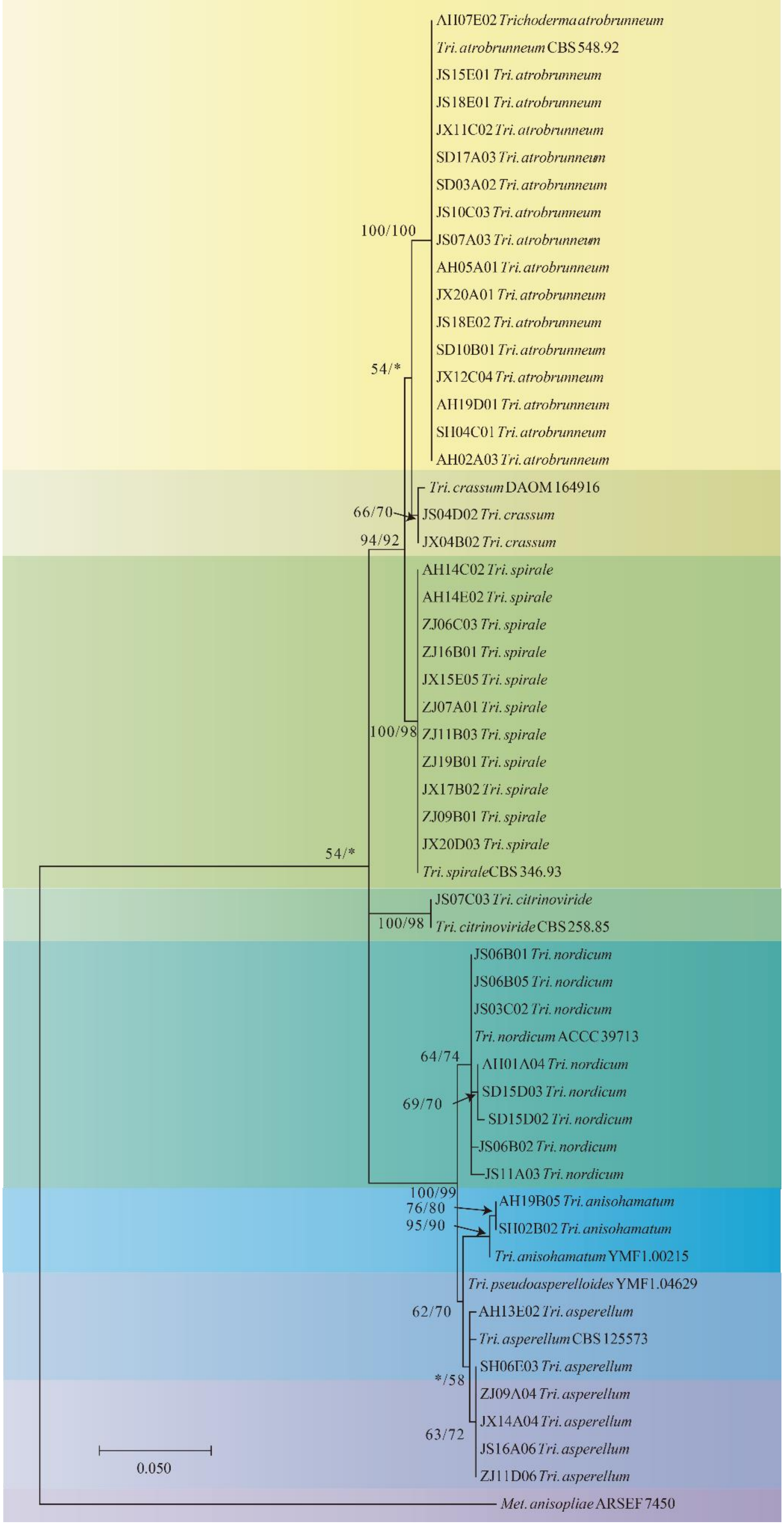

Figure S3.8 Phylogenetic tree of *Trichoderma* based on ITS gene

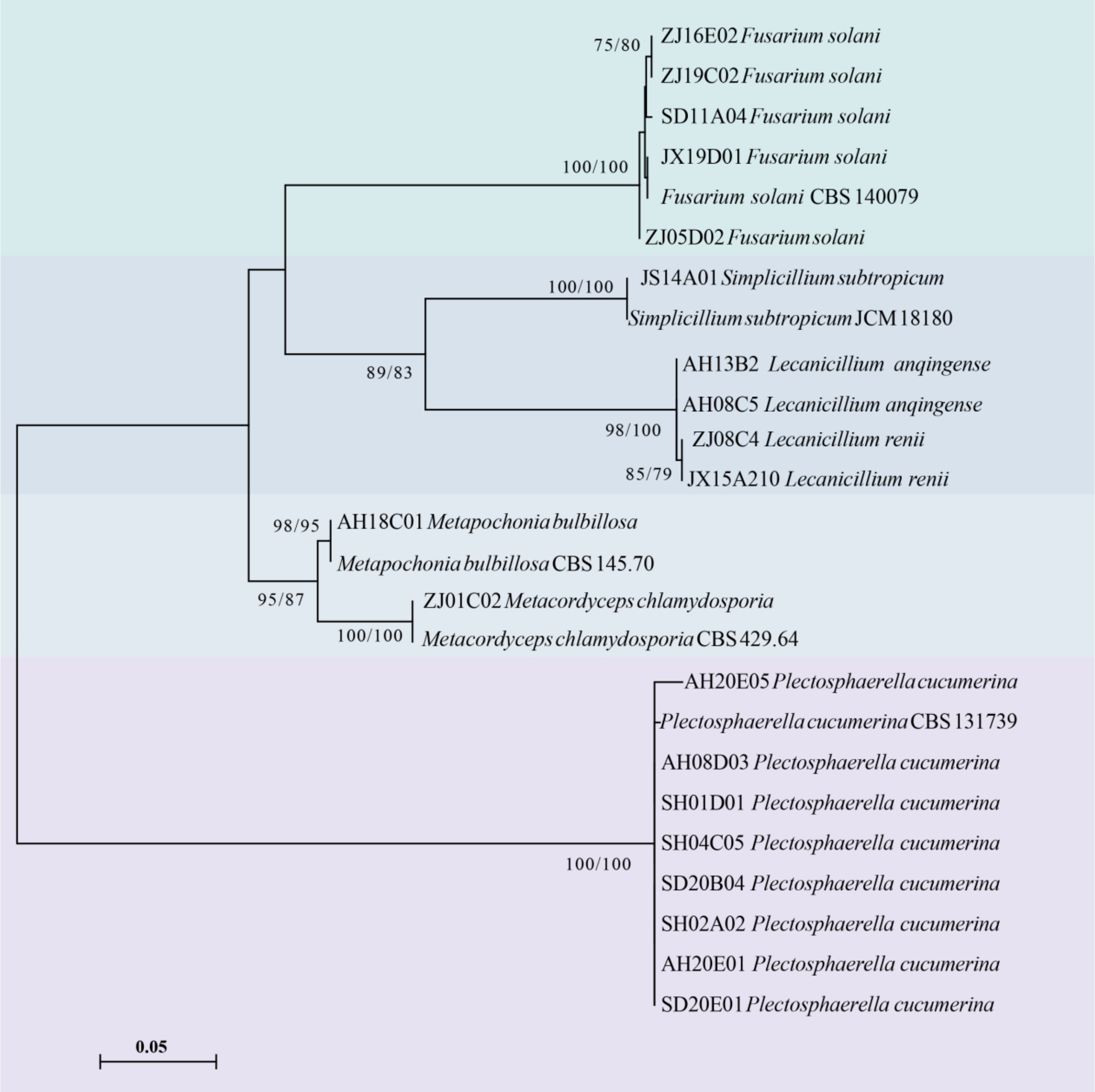

Figure S3.9 Phylogenetic tree of other genus based on ITS gene

sp
