# Supplementary material for: Biodiversity of entomopathogenic fungi in soils of eastern China
Source: Microbiol Spectr. 2026 Feb 13;14(4):e02904-25. doi: 10.1128/spectrum.02904-25 (PMC13055385; doi:10.1128/spectrum.02904-25)
Supplement: TABLE S1 — Detailed strain information. [file spectrum.02904-25-s0004.pdf]

Table S1 EGF strain information in detail

sample	Location	habitat	Strain	Species	Genebank NO.	
					ITS	LSU
AH01	Qiaocheng District,Bozhou City (33.9896°N, 115.6699°E)	Crop	AH01A01	*Pur. lavendulum*	PP384584	PP380638
		Forest	AH01B03	*Pur. lilacinum*	PP384590	PP380644
		Forest	AH01B04	*Pur. lilacinum*	PP384591	PP380645
		Orchard	AH01D02	*Pen. citrinum*	PP384593	PP380647
		Orchard	AH01D03	*Asp. ochraceus*	PP384594	PP380648
		Orchard	AH01D04	*Pen. citrinum*	PP384595	PP380649
AH02	Taihe County,Fuyang City (33.1696°N, 115.5980°E)	Crop	AH02A01	*Pen. citrinum*	PP384597	PP380651
		Forest	AH02B02	*Pur. lilacinum*	PP384600	PP380654
		Grass	AH02C02	*Pur. lavendulum*	PP384602	PP380656
		Grass	AH02C03	*Met. anisopliae*	PP384603	PP380657
		Fallow	AH02E01	*Pen. citrinum*	PP384607	PP380661
AH03	Huoqiu County,Lu'an City (31.3707°N, 116.1937°E)	Forest	AH03B01	*Pen. citrinum*	PP384612	PP380666
		Forest	AH03B02	*Pur. lilacinum*	PP384613	PP380667
		Forest	AH03B03	*Pen. guanacastense*	PP384614	PP380668
AH04	Sanhe Town,Huainan City (32.6091°N, 116.9536°E)	Crop	AH04A02	*Pur. lilacinum*	PP384617	PP380671
AH05	Huaiyuan County,Bengbu City (33.1367°N, 116.8915°E)	Forest	AH05B02	*Pen. citrinum*	PP384624	PP380678
		Forest	AH05B03	*Pen. citrinum*	PP384625	PP380679
		Fallow	AH05E01	*Pur. lilacinum*	PP384628	PP380682
		Fallow	AH05E02	*Cha. cochliodes*	PP384629	PP380683
		Fallow	AH05E03	*Pur. lilacinum*	PP384630	PP380684
AH06	Woyang County, Bozhou City (33.4752°N, 116.4818°E)	Grass	AH06C03	*Pen. citrinum*	PP384633	PP380687
		Orchard	AH06D02	*Pur. lilacinum*	PP384636	PP380690
		Orchard	AH06D03	*Asp. flavus*	PP384637	PP380691
		Orchard	AH06D04	*Pen. citrinum*	PP384638	PP380692
AH07	Xiao County,Suzhou City (34.0920°N, 116.8734°E)	Forest	AH07B01	*Asp. insuetus*	PP384641	PP380695
		Orchard	AH07D02	*Met. gaoligongense*	PP384645	PP380699
		Orchard	AH07D05	*Met. brunneum*	PP384648	PP380702
		Orchard	AH07D06	*Bea. bassiana*	PP384649	PP380703
		Fallow	AH07E01	*Pen. citrinum*	PP384650	PP380704
AH08	Lingbi County, Suzhou City (33.5445°N, 117.5353°E)	Forest	AH08B02	*Asp. flavus*	PP384653	PP380707
		Forest	AH08B03	*Pur. lilacinum*	PP384654	PP380708
		Grass	AH08C01	*Pen. citrinum*	PP384655	PP380709
		Grass	AH08C02	*Bea. bassiana*	PP384656	PP380710
		Grass	AH08C05	*Lec. anqingense*	PP770679	PP770681
		Orchard	AH08D03	*Ple. cucumerina*	PP384658	PP380712
AH09	Suxiang Town, Chuzhou City (32.8717°N, 118.0966°E)	Fallow	AH09E01	*Met. anisopliae*	PP384663	PP380717
		Fallow	AH09E02	*Pur. lilacinum*	PP384664	PP380718
		Fallow	AH09E03	*Met. anisopliae*	PP384665	PP380719
AH10	Shiji Town, Chuzhou City (32.2823°N, 118.1205°E)	Forest	AH10B03	*Mar. marquandii*	PP384669	PP380723
		Orchard	AH10D03	*Pur. lilacinum*	PP384674	PP380728
		Orchard	AH10D04	*Cha. globosum*	PP384675	PP380729
		Fallow	AH10E02	*Pur. lilacinum*	PP384677	PP380731
AH11	Yaohai District, Hefei City (31.8815°N, 117.3614°E)	Orchard	AH11D01	*Pur. lilacinum*	PP384682	PP380736
		Orchard	AH11D02	*Pen. citrinum*	PP384683	PP380737
		Fallow	AH11E01	*Cha. cochliodes*	PP384685	PP380739
		Fallow	AH11E03	*Pur. lilacinum*	PP384687	PP380741
AH12	Wuwei City, Wuhu City (31.4146°N, 117.7307°E)	Crop	AH12A02	*Pen. citrinum*	PP384689	PP380743
		Forest	AH12B01	*Pen. guanacastense*	PP384692	PP380746
		Forest	AH12B04	*Pur. lilacinum*	PP384695	PP380749
		Grass	AH12C02	*Pur. lilacinum*	PP384697	PP380751
AH13	Tongcheng City, Anqing City (31.2315°N, 117.0261°E)	Crop	AH13A02	*Pen. citrinum*	PP384703	PP380757
		Forest	AH13B2	*Lec. anqingense*	PP647368	PP647554
		Grass	AH13C02	*Pur. lilacinum*	PP384706	PP380760
		Grass	AH13C03	*Pur. lilacinum*	PP384707	PP380761
		Orchard	AH13D01	*Pen. citrinum*	PP384709	PP380763
		Fallow	AH13E02	*Tri. asperellum*	PP384711	PP380765
AH14	Huoshan County, Luan City (32.3047°N, 116.1422°E)	Grass	AH14C01	*Pur. lilacinum*	PP384714	PP380768
		Grass	AH14C02	*Tri. spirale*	PP384715	PP380769
		Fallow	AH14E01	*Pen. daleae*	PP384718	PP380772
		Fallow	AH14E02	*Tri. spirale*	PP384719	PP380773
AH15	Taihu County, Anqing City (30.4291°N, 116.4274°E)	Grass	AH15C01	*Pur. lilacinum*	PP384722	PP380776
AH16	Shitai County, Chizhou City	Crop	AH16A03	*Clo. chloroleuca*	PP384728	PP380782

	(30.1912°N, 117.3530°E)					
		Grass	AH16C02	*Met. anisopliae*	PP384734	PP380788
		Orchard	AH16D01	*Pur. lilacinum*	PP384735	PP380789
		Orchard	AH16D04	*Asp. ochraceus*	PP384738	PP380792
		Fallow	AH16E02	*Met. anisopliae*	PP384740	PP380794
		Fallow	AH16E03	*Pur. lilacinum*	PP384741	PP380795
AH17	Huangshan District, Huangshan City (30.1648°N, 118.1254°E)	Grass	AH17C03	*Met. anisopliae*	PP384752	PP380806
		Grass	AH17C04	*Pur. lilacinum*	PP384753	PP380807
		Orchard	AH17D02	*Met. anisopliae*	PP384756	PP380810
		Fallow	AH17E01	*Met. gaoligongense*	PP384757	PP380811
AH18	Nanling County, Wuhu City (30.7217°N, 118.1655°E)	Crop	AH18A02	*Mar. marquandii*	PP384760	PP380814
		Grass	AH18C01	*Met. bulbillosa*	PP384767	PP380821
		Grass	AH18C02	*Pur. lilacinum*	PP384768	PP380822
		Orchard	AH18D02	*Pur. lilacinum*	PP384770	PP380824
		Fallow	AH18E03	*Pur. lilacinum*	PP384775	PP380829
AH19	Dangtu County, Maanshan City (31.4399°N, 118.6055°E)	Forest	AH19B02	*Pur. lilacinum*	PP384777	PP380831
		Forest	AH19B03	*Met. anisopliae*	PP384778	PP380832
		Forest	AH19B04	*Met. anisopliae*	PP384779	PP380833
		Grass	AH19C02	*Pur. lilacinum*	PP384782	PP380836
AH20	Ningguo City, Xuancheng City (30.6845°N, 118.9830°E)	Crop	AH20A01	*Pur. lilacinum*	PP384788	PP380842
		Crop	AH20A02	*Clo. chloroleuca*	PP384789	PP380843
		Crop	AH20A04	*Pur. lilacinum*	PP384791	PP380845
		Forest	AH20B01	*Pur. lilacinum*	PP384792	PP380846
		Forest	AH20B02	*Pur. lilacinum*	PP384793	PP380847
		Grass	AH20C01	*Pen. citrinum*	PP384796	PP380850
		Orchard	AH20D03	*Pur. lilacinum*	PP384800	PP380854
		Fallow	AH20E01	*Ple. cucumerina*	PP384803	PP380857
		Fallow	AH20E05	*Ple. cucumerina*	PP384806	PP380860
JS01	Haimen District, Nantong City (32.0332°N, 121.2715°E)	Crop	JS01A03	*Pur. lilacinum*	PP384809	PP380863
		Crop	JS01A04	*Mar. marquandii*	PP384810	PP380864
		Crop	JS01A06	*Met. robertsii*	PP384812	PP380866
		Fallow	JS01E02	*Met. gaoligongense*	PP384814	PP380868
JS02	Hai'an City, Nantong City (32.5572°N, 120.6817°E)	Crop	JS02A01	*Met. robertsii*	PP384815	PP380869
		Forest	JS02B01	*Pur. lilacinum*	PP384817	PP380871
		Forest	JS02B02	*Bea. bassiana*	PP384818	PP380872
		Forest	JS02B04	*Asp. fumigatus*	PP384820	PP380874
		Forest	JS02B05	*Pen. citrinum*	PP384821	PP380875
		Grass	JS02C01	*Asp. insuetus*	PP384822	PP380876
		Grass	JS02C02	*Pur. lilacinum*	PP384823	PP380877
		Orchard	JS02D03	*Asp. insuetus*	PP384826	PP380880
JS03	Taicang City, Suzhou City (31.6222°N, 121.2075°E)	Orchard	JS03D01	*Pur. lilacinum*	PP384832	PP380886
		Orchard	JS03D02	*Pur. lilacinum*	PP384833	PP380887
		Orchard	JS03D03	*Met. anisopliae*	PP384834	PP380888
JS04	Wuzhong District, Suzhou City (31.2827°N, 120.3933°E)	Crop	JS04A01	*Pur. lilacinum*	PP384838	PP380892
		Forest	JS04B02	*Pen. citrinum*	PP384840	PP380894
		Grass	JS04C03	*Pen. citrinum*	PP384844	PP380898
JS05	Jiangyin City, Wuxi City (31.8414°N, 120.2027°E)	Crop	JS05A04	*Pur. lilacinum*	PP384851	PP380905
		Forest	JS05B01	*Pur. lilacinum*	PP384852	PP380906
		Forest	JS05B02	*Bea. bassiana*	PP384853	PP380907
JS06	Danyang City, Zhenjiang City (32.0632°N, 119.5066°E)	Grass	JS06C02	*Pur. lilacinum*	PP384863	PP380917
		Orchard	JS06D01	*Asp. insuetus*	PP384864	PP380918
		Orchard	JS06D02	*Pen. citrinum*	PP384865	PP380919
		Fallow	JS06E01	*Pur. lilacinum*	PP384867	PP380921
JS07	Liyang City, Changzhou City (31.5736°N, 119.3861°E)	Crop	JS07A01	*Pen. citrinum*	PP384869	PP380923
		Crop	JS07A02	*Cha. cochliodes*	PP384870	PP380924
		Grass	JS07C03	*Tri. Citrinoviride*	PP384874	PP380928
		Grass	JS07C04	*Pur. lilacinum*	PP384875	PP380929
JS08	Jiangning District, Nanjing City (31.7447°N, 118.7163°E)	Forest	JS08B02	*Pur. lilacinum*	PP384879	PP380933
		Forest	JS08B03	*Pen. brefeldianum*	PP384880	PP380934
JS09	Luhe District, Nanjing City (32.4626°N, 118.8032°E)	Grass	JS09C02	*Mar. marquandii*	PP384889	PP380943
		Orchard	JS09D01	*Pur. lilacinum*	PP384890	PP380944
		Orchard	JS09D02	*Pur. lilacinum*	PP384891	PP380945
JS10	Xuyi County, Huai'an City (33.0815°N, 118.7414°E)	Forest	JS10B01	*Pur. lilacinum*	PP384896	PP380950
		Forest	JS10B02	*Met. anisopliae*	PP384897	PP380951
		Forest	JS10B04	*Pur. lilacinum*	PP384899	PP380953
		Orchard	JS10D02	*Pur. lilacinum*	PP384905	PP380959
JS11	Baoying County, Yangzhou City (33.2790°N, 119.4151°E)	Crop	JS11A04	*Pur. lilacinum*	PP384912	PP380966

		Forest	JS11B01	*Clo. chloroleuca*	PP384915	PP380969
		Grass	JS11C01	*Pen. citrinum*	PP384916	PP380970
		Grass	JS11C02	*Pur. lilacinum*	PP384917	PP380971
		Fallow	JS11E01	*Met. anisopliae*	PP384922	PP380976
		Fallow	JS11E02	*Pur. lilacinum*	PP384923	PP380977
		Fallow	JS11E03	*Asp. flavus*	PP384924	PP380978
		Fallow	JS11E04	*Met. anisopliae*	PP384925	PP380979
JS12	Xinghua City, Taizhou City (32.9612°N, 119.8934°E)	Forest	JS12B02	*Pen. citrinum*	PP384927	PP380981
		Grass	JS12C01	*Bea. bassiana*	PP384928	PP380982
		Grass	JS12C02	*Pur. lilacinum*	PP384929	PP380983
		Orchard	JS12D01	*Pur. lilacinum*	PP384930	PP380984
		Fallow	JS12E02	*Pur. lilacinum*	PP384933	PP380987
JS13	Dafeng District, Yancheng City (33.2010°N, 120.5462°E)	Forest	JS13B01	*Pur. lilacinum*	PP384938	PP380992
		Forest	JS13B02	*Pur. lilacinum*	PP384939	PP380993
		Forest	JS13B03	*Met. anisopliae*	PP384940	PP380994
		Forest	JS13B04	*Met. robertsii*	PP384941	PP380995
		Forest	JS13B05	*Pur. lavendulum*	PP384942	PP380996
		Fallow	JS13E01	*Met. robertsii*	PP384945	PP380999
		Fallow	JS13E02	*Met. robertsii*	PP384946	PP381000
		Fallow	JS13E03	*Met. brunneum*	PP384947	PP381001
JS14	Funing County, Yancheng City (33.7600°N, 119.6567°E)	Crop	JS14A01	*Sim. subtropicum*	PP384948	PP381002
		Crop	JS14A05	*Met. robertsii*	PP384952	PP381006
		Forest	JS14B01	*Pur. lilacinum*	PP384955	PP381009
		Orchard	JS14D01	*Pur. lilacinum*	PP384960	PP381014
JS15	Binhai County, Yancheng City (34.1494°N, 120.0932°E)	Grass	JS15C01	*Pur. lilacinum*	PP384966	PP381020
		Grass	JS15C02	*Pur. lilacinum*	PP384967	PP381021
		Orchard	JS15D01	*Met. anisopliae*	PP384969	PP381023
		Orchard	JS15D02	*Mar. marquandii*	PP384970	PP381024
JS16	Donghai County, Lianyungang City (34.7095°N, 118.9243°E)	Crop	JS16A04	*Pur. lilacinum*	PP384976	PP381030
		Crop	JS16A05	*Pen. citrinum*	PP384977	PP381031
		Crop	JS16A06	*Tri. asperellum*	PP384978	PP381032
JS17	Shuyang County, Suqian City (34.0691°N, 119.0225°E)	Forest	JS17B01	*Pur. lilacinum*	PP384985	PP381039
		Grass	JS17C01	*Pur. lilacinum*	PP384987	PP381041
		Grass	JS17C02	*Pur. lavendulum*	PP384988	PP381042
JS19	Tongshan District, Xuzhou City (34.1499°N, 117.5261°E)	Crop	JS19A01	*Met. robertsii*	PP384992	PP381046
		Grass	JS19C04	*Met. robertsii*	PP384997	PP381051
		Orchard	JS19D03	*Cha. globosum*	PP385000	PP381054
		Fallow	JS19E01	*Pur. lilacinum*	PP385004	PP381058
		Fallow	JS19E03	*Bea. bassiana*	PP385006	PP381060
JS20	Xinyi City, Xuzhou City (34.3597°N, 118.3366°E)	Crop	JS20A01	*Pen. citrinum*	PP385007	PP381061
		Fallow	JS20E02	*Pur. lilacinum*	PP385015	PP381069
JX02	Chongyi County, Ganzhou City (25.7164°N, 114.3147°E)	Forest	JX02B01	*Pur. lilacinum*	PP385018	PP381072
		Forest	JX02B02	*Pur. lilacinum*	PP385019	PP381073
JX03	Huichang County, Ganzhou City (25.7348°N, 115.6618°E)	Grass	JX03C01	*Pur. lilacinum*	PP385021	PP381075
		Grass	JX03C02	*Pur. lilacinum*	PP385022	PP381076
		Orchard	JX03D01	*Pur. lilacinum*	PP385023	PP381077
JX04	Xingguo County, Ganzhou City (26.5423°N, 115.3507°E)	Crop	JX04A04	*Pur. lilacinum*	PP385027	PP381081
		Crop	JX04A07	*Pur. lilacinum*	PP385030	PP381084
		Forest	JX04B01	*Pur. lilacinum*	PP385031	PP381085
JX05	Wan'an County, Ji'an City (26.4136°N, 114.7806°E)	Crop	JX05A02	*Pur. lilacinum*	PP385036	PP381090
JX06	Yongxin County, Ji'an City (27.0423°N, 114.2621°E)	Grass	JX06C01	*Pen. janthinellum*	PP385037	PP381091
		Grass	JX06C03	*Pur. lilacinum*	PP385039	PP381093
		Grass	JX06C05	*Pur. lilacinum*	PP385041	PP381095
		Grass	JX06C06	*Asp. aureoterreus*	PP385042	PP381096
JX08	Shangli County, Pingxiang City (27.6987°N, 113.8297°E)	Fallow	JX08E02	*Pur. lilacinum*	PP385044	PP381098
		Fallow	JX08E03	*Pur. lilacinum*	PP385045	PP381099
JX09	Yifeng County, Yichun City (28.3891°N, 114.7672°E)	Crop	JX09A03	*Pur. lilacinum*	PP385047	PP381101
		Crop	JX09A04	*Pur. lilacinum*	PP385048	PP381102
JX11	Pengze County, Jiujiang City (29.7943°N, 116.5754°E)	Crop	JX11A01	*Pen. citrinum*	PP385052	PP381106
		Crop	JX11A02	*Pen. citrinum*	PP385053	PP381107
		Crop	JX11A04	*Pur. lilacinum*	PP385054	PP381108
		Forest	JX11B01	*Clo. chloroleuca*	PP385056	PP381110
		Grass	JX11C01	*Mar. marquandii*	PP385057	PP381111
		Orchard	JX11D01	*Asp. flavus*	PP385060	PP381114
		Orchard	JX11D03	*Clo. chloroleuca*	PP385062	PP381116
		Orchard	JX11D04	*Met. robertsii*	PP385063	PP381117
		Fallow	JX11E05	*Clo. chloroleuca*	PP385068	PP381122

JX12	Poyang County, Shangrao City	Grass	JX12C06	*Pur. lilacinum*	PP385077	PP381131
	(29.0714°N, 116.7042°E)	Orchard	JX12D02	*Cha. cochliodes*	PP385079	PP381133
		Fallow	JX12E01	*Pur. lilacinum*	PP385082	PP381136
JX13	Meiling Town, Nanchang City	Crop	JX13A03	*Pur. lilacinum*	PP385085	PP381139
	(28.7913°N, 115.7551°E)					
		Crop	JX13A04	*Pur. lilacinum*	PP385086	PP381140
		Crop	JX13A05	*Asp. flavus*	PP385087	PP381141
		Crop	JX13A06	*Met. anisopliae*	PP385088	PP381142
		Forest	JX13B01	*Pur. Jiangxiense*	PP555637	PP555646
		Fallow	JX13E04	*Asp. aureoterreus*	PP385094	PP381148
JX14	Xingan County, Ji'an City	Crop	JX14A03	*Pen. citrinum*	PP385097	PP381151
	(27.8349°N, 115.4733°E)					
		Crop	JX14A04	*Tri. asperellum*	PP385098	PP381152
		Crop	JX14A05	*Pur. lilacinum*	PP385099	PP381153
		Forest	JX14B03	*Pen. citrinum*	PP385102	PP381156
JX15	Ningdu County, Ganzhou City	Crop	JX15A02	*Pur. lilacinum*	PP385106	PP381160
	(26.9168°N, 116.0076°E)					
		Crop	JX15A03	*Pen. brefeldianum*	PP385107	PP381161
		Crop	JX15A06	*Pen. brefeldianum*	PP385109	PP381163
		Crop	JX15A210	*Lec. renii*	PP647368	PP647552
		Forest	JX15B01	*Pur. lilacinum*	PP385110	PP381164
		Forest	JX15B05	*Met. robertsii*	PP385113	PP381167
		Grass	JX15C01	*Pur. lilacinum*	PP385114	PP381168
		Grass	JX15C02	*Pen. citrinum*	PP385115	PP381169
		Orchard	JX15D01	*Pen. citrinum*	PP385116	PP381170
		Fallow	JX15E03	*Pen. citrinum*	PP385121	PP381175
		Fallow	JX15E04	*Pur. lilacinum*	PP385122	PP381176
		Fallow	JX15E05	*Tri. spirale*	PP385123	PP381177
JX16	Linchuan District, Fuzhou City	Forest	JX16B03	*Pen. brefeldianum*	PP385126	PP381180
	(27.7091°N, 116.2889°E)					
		Grass	JX16C02	*Pur. lilacinum*	PP385128	PP381182
		Grass	JX16C04	*Pur. lilacinum*	PP385130	PP381184
		Grass	JX16E03	*Pen. citrinum*	PP385133	PP381187
JX17	Lichuan County, Fuzhou City	Forest	JX17B02	*Tri. spirale*	PP385141	PP381195
	(27.3591°N, 116.9866°E)					
		Forest	JX17B03	*Pur. lilacinum*	PP385142	PP381196
		Orchard	JX17D05	*Pur. lilacinum*	PP385149	PP381203
		Orchard	JX17D06	*Pen. citrinum*	PP385150	PP381204
		Fallow	JX17E01	*Met. anisopliae*	PP385151	PP381205
		Fallow	JX17E02	*Pur. lilacinum*	PP385152	PP381206
		Crop	JX18A01	*Pen. janthinellum*	PP385153	PP381207
JX18	Guixi City, Yingtan City					
	(28.2846°N, 117.2101°E)	Crop	JX18A03	*Clo. chloroleuca*	PP385155	PP381209
		Crop	JX18B02	*Pur. lilacinum*	PP385157	PP381211
		Forest	JX18E04	*Asp. aureoterreus*	PP385167	PP381221
		Crop	JX19A01	*Pur. lilacinum*	PP385170	PP381224
JX19	Dexing City, Shangrao City					
	(29.0213°N, 117.6408°E)	Crop	JX19A02	*Pen. janthinellum*	PP385171	PP381225
		Forest	JX19B01	*Pur. lilacinum*	PP385173	PP381227
		Orchard	JX19D01	*Fus. solani*	PP385177	PP381231
		Orchard	JX19D02	*Pur. lilacinum*	PP385178	PP381232
		Crop	JX20A03	*Pen. citrinum*	PP385183	PP381237
JX20	Guangfeng District, Shangrao City					
	(28.3824°N, 118.2482°E)	Crop	JX20A05	*Pur. lilacinum*	PP385185	PP381239
		Grass	JX20C04	*Pur. lilacinum*	PP385194	PP381248
		Orchard	JX20D03	*Tri. spirale*	PP385197	PP381251
		Crop	SD01A02	*Pen. citrinum*	PP385200	PP381254
SD01	Xinzhuang Town, Linyi City					
	(35.0963°N, 117.8722°E)	Fallow	SD01E01	*Pur. lilacinum*	PP385206	PP381260
		Fallow	SD01E02	*Pur. lilacinum*	PP385207	PP381261
		Fallow	SD01E03	*Pur. lilacinum*	PP385208	PP381262
		Forest	SD02B01	*Asp. flavus*	PP385213	PP381267
SD02	Tengzhou City, Zaozhuang City					
	(35.1346°N, 116.9050°E)	Forest	SD02B02	*Pen. guanacastense*	PP385214	PP381268
		Forest	SD02B04	*Pen. citrinum*	PP385216	PP381270
		Forest	SD03B01	*Pur. lilacinum*	PP385222	PP381276
SD03	Ningyang County, Tai'an City					
	(35.7655°N, 116.7703°E)	Forest	SD03B02	*Pur. lilacinum*	PP385223	PP381277
		Orchard	SD03D01	*Pen. citrinum*	PP385226	PP381280
		Orchard	SD03D02	*Pen. guanacastense*	PP385227	PP381281
		Crop	SD04A01	*Pen. citrinum*	PP385230	PP381284
SD04	Taishan District, Tai'an City					
	(36.2988°N, 117.1411°E)	Crop	SD04A03	*Met. anisopliae*	PP385231	PP381285
		Forest	SD04B02	*Pur. lilacinum*	PP385233	PP381287
		Grass	SD04C03	*Pur. lavendulum*	PP385236	PP381290
		Grass	SD04C04	*Pur. lilacinum*	PP385237	PP381291
		Crop	SD05A01	*Pur. lilacinum*	PP385242	PP381296
SD05	Sishui County, Jining City					
	(35.6527°N, 117.3957°E)	Crop	SD05A03	*Pur. lilacinum*	PP385244	PP381298
		Crop	SD05A04	*Bea. bassiana*	PP385245	PP381299
		Forest	SD05B01	*Met. baoshanense*	PP385246	PP381300
		Orchard	SD05D03	*Met. anisopliae*	PP385249	PP381303

		Crop	SD06A01	*Pur. lilacinum*	PP385251	PP381305
SD06	Yishui County, Linyi City					
	(35.8226°N, 118.3220°E)	Grass	SD06C01	*Pur. lilacinum*	PP385253	PP381307
		Orchard	SD06D05	*Pur. lilacinum*	PP385259	PP381313
		Forest	SD08B01	*Pur. lilacinum*	PP385273	PP381327
SD08	Anqiu City, Weifang City					
	(36.3108°N, 119.3916°E)	Orchard	SD08D02	*Pur. lilacinum*	PP385279	PP381333
		Orchard	SD08D03	*Pur. lilacinum*	PP385280	PP381334
		Crop	SD09A03	*Pur. lilacinum*	PP385286	PP381340
SD09	Laixi City, Qingdao City					
	(37.0096°N, 120.5794°E)	Grass	SD09C01	*Pur. lilacinum*	PP385288	PP381342
		Crop	SD09C02	*Pen. citrinum*	PP385289	PP381343
		Fallow	SD09E01	*Pur. lilacinum*	PP385293	PP381347
		Fallow	SD10E02	*Pur. lilacinum*	PP385307	PP381361
SD10	Muping District, Yantai City	Fallow				
	(37.1697°N, 121.4075°E)	Fallow	SD10E03	*Met. anisopliae*	PP385308	PP381362
		Crop	SD11A02	*Cha. cochliodes*	PP385310	PP381364
SD11	Hanting District, Weifang City					
	(36.8452°N, 119.0428°E)	Crop	SD11A03	*Pen. citrinum*	PP385311	PP381365
		Crop	SD11A04	*Fus. solani*	PP385312	PP381366
		Crop	SD11A05	*Met. robertsii*	PP385313	PP381367
		Crop	SD11A06	*Pur. lilacinum*	PP385314	PP381368
		Crop	SD11A07	*Met. robertsii*	PP385315	PP381369
		Forest	SD11B01	*Pur. lilacinum*	PP385316	PP381370
		Fallow	SD11E02	*Bea. bassiana*	PP385319	PP381373
		Fallow	SD11E03	*Mar. marquandii*	PP385320	PP381374
		Fallow	SD11E04	*Pur. lilacinum*	PP385321	PP381375
		Crop	SD12A05	*Met. robertsii*	PP385326	PP381380
SD12	Kenli District, Dongying City					
	(37.6875°N, 118.8400°E)	Forest	SD12B01	*Pen. citrinum*	PP385328	PP381382
		Forest	SD12B02	*Pur. lilacinum*	PP385329	PP381383
		Forest	SD12B03	*Pur. lilacinum*	PP385330	PP381384
		Grass	SD12C03	*Met. robertsii*	PP385334	PP381388
		Fallow	SD12E02	*Pur. lilacinum*	PP385338	PP381392
		Crop	SD13A02	*Met. anisopliae*	PP385342	PP381396
SD13	Wudi County, Binzhou City					
	(37.9950°N, 117.8828°E)	Forest	SD13B02	*Pur. lilacinum*	PP385344	PP381398
		Forest	SD13B05	*Met. robertsii*	PP385347	PP381401
		Grass	SD13C03	*Met. anisopliae*	PP385350	PP381404
		Grass	SD13C04	*Pur. lilacinum*	PP385351	PP381405
		Orchard	SD13D01	*Pur. lilacinum*	PP385352	PP381406
		Orchard	SD13D03	*Pur. lilacinum*	PP385354	PP381408
		Fallow	SD13E03	*Met. anisopliae*	PP385358	PP381412
		Fallow	SD13E04	*Pur. lilacinum*	PP385359	PP381413
		Crop	SD14A02	*Pur. lilacinum*	PP385363	PP381417
SD14	Ningjin County, Dezhou City					
	(37.7050°N, 116.9537°E)	Forest	SD14B01	*Pur. lilacinum*	PP385364	PP381418
		Forest	SD14B02	*Met. robertsii*	PP385365	PP381419
		Forest	SD14B03	*Pur. lilacinum*	PP385366	PP381420
		Grass	SD14C01	*Pen. sizovae*	PP385370	PP381424
		Fallow	SD14E02	*Pur. lilacinum*	PP385377	PP381431
		Crop	SD15A01	*Met. anisopliae*	PP385378	PP381432
SD15	Jiyang District, Jinan City					
	(37.1648°N, 117.3647°E)	Grass	SD15C04	*Cha. cochliodes*	PP385384	PP381438
		Grass	SD15C05	*Pur. lilacinum*	PP385385	PP381439
		Orchard	SD15D01	*Pur. lilacinum*	PP385388	PP381442
		Orchard	SD15D04	*Pur. lilacinum*	PP385391	PP381445
		Fallow	SD15E01	*Pur. lilacinum*	PP385392	PP381446
		Forest	SD16B02	*Pur. lilacinum*	PP385397	PP381451
SD16	Zhangdian District, Zibo City					
	(36.7813°N, 118.0200°E)	Forest	SD16B03	*Pur. lilacinum*	PP385398	PP381452
		Forest	SD16B05	*Pur. lavendulum*	PP385400	PP381454
		Forest	SD16B07	*Pur. lilacinum*	PP385402	PP381456
			SD16E01	*Pur. lilacinum*	PP385404	PP381458
		Fallow	SD16E02	*Pen. citrinum*	PP385405	PP381459
		Crop	SD17A01	*Pur. lilacinum*	PP385406	PP381460
SD17	Yucheng City, Dezhou City					
	(36.8096°N, 116.4287°E)	Crop	SD17A02	*Met. anisopliae*	PP385407	PP381461
		Forest	SD17B02	*Pur. lilacinum*	PP385410	PP381464
		Forest	SD17B04	*Met. baoshanense*	PP385412	PP381466
		Grass	SD17C01	*Pur. lilacinum*	PP385413	PP381467
		Grass	SD17D02	*Pur. lilacinum*	PP385416	PP381470
		Orchard	SD17E01	*Pur. lilacinum*	PP385420	PP381474
		Fallow	SD17E02	*Met. robertsii*	PP385421	PP381475
		Grass	SD18C02	*Pur. lilacinum*	PP385427	PP381481
SD18	Shen County, Liaocheng City	Grass	SD18C03	*Pur. lilacinum*	PP385428	PP381482
	(36.3115°N, 115.5178°E)	Fallow	SD18E02	*Pen. citrinum*	PP385435	PP381489
		Crop	SD19A02	*Asp. flavus*	PP385436	PP381490
SD19	Yuncheng County, Heze City					
	(35.4892°N, 115.8412°E)	Fallow	SD19E01	*Pur. lilacinum*	PP385441	PP381495
		Forest	SD20B01	*Pen. citrinum*	PP385446	PP381500
SD20	Shanxian County, Heze City					

	(34.7352°N, 115.9271°E)	Forest	SD20B04	*Ple. cucumerina*	PP385449	PP381503
		Orchard	SD20D01	*Pur. lilacinum*	PP385451	PP381505
		Fallow	SD20E01	*Ple. cucumerina*	PP385454	PP381508
		Fallow	SD20E02	*Pur. lilacinum*	PP385455	PP381509
		Fallow	SD20E03	*Pur. lilacinum*	PP385456	PP381510
		Fallow	SD20E04	*Pur. lilacinum*	PP385457	PP381511
		Crop	SH01A01	*Pur. lilacinum*	PP385458	PP381512
SH01	Jinshan District, Shanghai					
	(121.2902°N,30.7360°E)	Crop	SH01A05	*Mar. marquandii*	PP385462	PP381516
		Grass	SH01C02	*Pen. citrinum*	PP385465	PP381519
		Orchard	SH01D01	*Ple. cucumerina*	PP385466	PP381520
		Crop	SH02A01	*Pur. lilacinum*	PP385467	PP381521
SH02	Fengxian District, Shanghai					
	(121.6394°N,30.9032°E)	Crop	SH02A02	*Ple. cucumerina*	PP385468	PP381522
		Crop	SH02A04	*Met. baoshanense*	PP385470	PP381524
		Forest	SH02B03	*Met. robertsii*	PP385473	PP381527
		Orchard	SH02D04	*Clo. chloroleuca*	PP385479	PP381533
		Crop	SH03A03	*Pur. lilacinum*	PP385482	PP381536
SH03	Pudong New Area, Shanghai					
	(121.5951°N,31.0329°E)	Forest	SH03B01	*Pur. lilacinum*	PP385484	PP381538
		Forest	SH03B03	*Pur. lilacinum*	PP385486	PP381540
		Forest	SH03B05	*Pen. citrinum*	PP385488	PP381542
		Crop	SH04A03	*Asp. flavus*	PP385492	PP381546
SH04	Chongming District, Shanghai					
	(121.7679°N,31.5987°E)	Forest	SH04B03	*Mar. marquandii*	PP385495	PP381549
		Grass	SH04C03	*Mar. marquandii*	PP385498	PP381552
		Grass	SH04C05	*Ple. cucumerina*	PP385500	PP381554
		Orchard	SH04D01	*Pen. citrinum*	PP385501	PP381555
		Crop	SH05A01	*Met. anisopliae*	PP385503	PP381557
SH05	Jiading District, Shanghai					
	(121.2678°N,31.3699°E)	Forest	SH05B01	*Pur. lilacinum*	PP385505	PP381559
		Forest	SH05B03	*Pur. lilacinum*	PP385507	PP381561
		Grass	SH05C01	*Met. brunneum*	PP385508	PP381562
		Orchard	SH05D01	*Mar. marquandii*	PP385509	PP381563
		Forest	SH06B02	*Pur. lilacinum*	PP385511	PP381565
SH06	Songjiang District, Shanghai					
	(121.2251°N,31.0171°E)	Forest	SH06B04	*Pur. lilacinum*	PP385513	PP381567
		Orchard	SH06D02	*Pen. citrinum*	PP385516	PP381570
		Fallow	SH06E03	*Tri. asperellum*	PP385519	PP381573
		Crop	SH07A03	*Pen. citrinum*	PP385521	PP381575
SH07	Qingpu District, Shanghai					
	(121.0277°N,31.1254°E)	Crop	SH07A05	*Pen. citrinum*	PP385523	PP381577
		Forest	SH07B02	*Pur. lavendulum*	PP385525	PP381579
		Forest	SH07B03	*Mar. marquandii*	PP385526	PP381580
		Grass	SH07C02	*Pen. oxalicum*	PP385528	PP381582
		Grass	SH07C03	*Pur. lilacinum*	PP385529	PP381583
		Crop	ZJ01A01	*Pen. citrinum*	PP385533	PP381587
ZJ01	Qujiang District, Quzhou City					
	(28.6785°N, 118.9355°E)	Forest	ZJ01B01	*Pur. lilacinum*	PP385534	PP381588
		Grass	ZJ01C02	*Poc. chlamydosporia*	PP385537	PP381591
		Fallow	ZJ01E02	*Pur. lilacinum*	PP385542	PP381596
		Fallow	ZJ01E03	*Pen. brefeldianum*	PP385543	PP381597
		Crop	ZJ02A02	*Pur. lilacinum*	PP385545	PP381599
ZJ02	Wuyi County, Jinhua City					
	(28.7146°N, 119.6016°E)	Forest	ZJ02B03	*Pen. citrinum*	PP385548	PP381602
		Grass	ZJ02C02	*Pur. lilacinum*	PP385549	PP381603
		Orchard	ZJ02D01	*Met. anisopliae*	PP385550	PP381604
		Crop	ZJ03A02	*Pur. lilacinum*	PP385555	PP381609
ZJ03	Qingtian County, Lishui City					
	(28.3140°N, 119.9766°E)	Forest	ZJ03B01	*Pen. citrinum*	PP385556	PP381610
		Grass	ZJ03C01	*Pen. janthinellum*	PP385557	PP381611
		Grass	ZJ03C03	*Pen. janthinellum*	PP385559	PP381613
		Crop	ZJ04A02	*Pur. lilacinum*	PP385563	PP381617
ZJ04	Longquan City, Lishui City					
	(28.0388°N, 119.1895°E)	Forest	ZJ04B01	*Pen. citrinum*	PP385564	PP381618
		Forest	ZJ04B02	*Pur. lavendulum*	PP385565	PP381619
		Crop	ZJ05A03	*Asp. flavus*	PP385574	PP381628
ZJ05	Taishun County, Wenzhou City					
	(27.6205°N, 119.9384°E)	Grass	ZJ05C02	*Pen. brefeldianum*	PP385576	PP381630
		Orchard	ZJ05D02	*Fus. solani*	PP385578	PP381632
		Crop	ZJ06A02	*Pen. citrinum*	PP385585	PP381639
ZJ06	Ruian City, Wenzhou City					
	(27.7562°N, 120.5106°E)	Grass	ZJ06C01	*Pur. lilacinum*	PP385590	PP381644
		Grass	ZJ06C03	*Tri. spirale*	PP385592	PP381646
		Crop	ZJ07A01	*Tri. spirale*	PP385597	PP381651
ZJ07	Yongjia County, Wenzhou City					
	(28.3894°N, 120.7268°E)	Crop	ZJ08A02	*Pen. janthinellum*	PP385608	PP381662
ZJ08	Huangyan District, Taizhou City					
	(28.5794°N, 121.0434°E)	Grass	ZJ08C01	*Pur. lilacinum*	PP385610	PP381664
		Grass	ZJ08C04	*Lec. renii*	PP770678	PP770680
		Fallow	ZJ08E02	*Pen. janthinellum*	PP385613	PP381667
		Crop	ZJ09A02	*Pen. citrinum*	PP385615	PP381669

ZJ09	Ninghai County, Ningbo City								
	(29.4868°N, 121.4492°E)	Crop	ZJ09A03	*Pen. janthinellum*	PP385616	PP381670			
		Crop	ZJ09A04	*Tri. asperellum*	PP385617	PP381671			
		Crop	ZJ09A05	*Pen. janthinellum*	PP385618	PP381672			
		Forest	ZJ09B01	*Tri. spirale*	PP385620	PP381674			
		Grass	ZJ10C02	*Pur. lilacinum*	PP385632	PP381686			
ZJ10	Yinzhou District, Ningbo City								
	(29.8167°N, 121.7412°E)	Fallow	ZJ10E03	*Pur. lilacinum*	PP385635	PP381689			
		Forest	ZJ11B03	*Tri. spirale*	PP385645	PP381699			
ZJ11	Putuo District, Zhoushan City								
	(29.9716°N, 122.2569°E)	Grass	ZJ11C03	*Pen. citrinum*	PP385648	PP381702			
		Orchard	ZJ11D02	*Pur. lilacinum*	PP385650	PP381704			
		Orchard	ZJ11D06	*Tri. asperellum*	PP385654	PP381708			
		Crop	ZJ12A04	*Pur. lilacinum*	PP385659	PP381713			
ZJ12	DaishanCounty, Zhoushan City								
	(30.2437°N, 122.2050°E)	Forest	ZJ12B01	*Pen. citrinum*	PP385661	PP381715			
		Forest	ZJ12B03	*Mar. marquandii*	PP385663	PP381717			
		Grass	ZJ12C01	*Pur. lavendulum*	PP385664	PP381718			
		Grass	ZJ12C02	*Pur. lilacinum*	PP385665	PP381719			
		Grass	ZJ12C03	*Pen. citrinum*	PP385666	PP381720			
		Orchard	ZJ12D01	*Pur. lavendulum*	PP385667	PP381721			
		Crop	ZJ13A01	*Pen. citrinum*	PP385669	PP381723			
ZJ13	Yuecheng District, Shaoxing City								
	(29.9473°N, 120.7417°E)	Orchard	ZJ13D01	*Pen. citrinum*	PP385671	PP381725			
		Orchard	ZJ13D06	*Pur. lilacinum*	PP385676	PP381730			
		Crop	ZJ14A03	*Pur. lilacinum*	PP385685	PP381739			
ZJ14	Xinchang County, Shaoxing City								
	(29.3932°N, 120.7823°E)	Crop	ZJ14A04	*Met. anisopliae*	PP385686	PP381740			
		Forest	ZJ14B01	*Pen. daleae*	PP385688	PP381742			
		Forest	ZJ14B03	*Met. anisopliae*	PP385690	PP381744			
		Crop	ZJ15A02	*Pur. lilacinum*	PP385695	PP381749			
ZJ15	Yiwu City, Jinhua City								
	(29.3707°N, 120.0568°E)	Orchard	ZJ15D02	*Asp. ochraceus*	PP385699	PP381753			
		Fallow	ZJ15E02	*Pen. citrinum*	PP385702	PP381756			
		Crop	ZJ16A01	*Pur. lilacinum*	PP385706	PP381760			
ZJ16	Jiande City, Hangzhou City								
	(29.6152°N, 119.5516°E)	Crop	ZJ16A03	*Pen. citrinum*	PP385708	PP381762			
		Forest	ZJ16B01	*Tri. spirale*	PP385709	PP381763			
		Orchard	ZJ16D04	*Pur. lilacinum*	PP385715	PP381769			
		Fallow	ZJ16E02	*Fus. solani*	PP385717	PP381771			
		Crop	ZJ17A04	*Pur. lilacinum*	PP385726	PP381780			
ZJ17	Chun'an County, Hangzhou City								
	(29.4007°N, 118.7389°E)	Crop	ZJ17A05	*Pur. lilacinum*	PP385727	PP381781			
		Forest	ZJ17B03	*Pen. daleae*	PP385730	PP381784			
		Crop	ZJ18A05	*Pur. lilacinum*	PP385739	PP381793			
ZJ18	Lin'an District, Hangzhou								
	(29.9996°N, 119.1358°E)	Grass	ZJ18C01	*Pen. janthinellum*	PP385742	PP381796			
		Orchard	ZJ18D01	*Pen. daleae*	PP385744	PP381798			
		Forest	ZJ19B01	*Tri. spirale*	PP385745	PP381799			
ZJ19	Fuyang District, Hangzhou City								
	(30.1530°N, 119.9847°E)	Grass	ZJ19C02	*Fus. solani*	PP385747	PP381801			
		Grass	ZJ19C05	*Pur. lilacinum*	PP385749	PP381803			
		Grass	ZJ20C03	*Asp. fumigatus*	PP385754	PP381808			
ZJ20	Anji County, Huzhou City								
	(30.6730°N, 119.6461°E)	Fallow	ZJ20E01	*Pur. lilacinum*	PP385757	PP381811			
ZJ21	Xiuzhou District, Jiaxing City (30.6681°N, 120.6280°E)	Orchard	ZJ21D01	*Pen. citrinum*	PP385768	PP381822			
				444	*444*				
**new species**					ITS	*Bloc*	*RPB1*	*tef1*	
AH08	Lingbi County, Suzhou City (33.5445°N, 117.5353°E)	orchard	AH08D02	*Beauveria* sp1	PP863318	PV659038	PV659043	PV659048	
JS02	Hai'an City, Nantong City (32.5572°N, 120.6817°E)	crop	JS02A02	*Beauveria* sp2	PP863319	PV659040	PV659045	PV659050	
SD18	Shen County, Liaocheng City (36.3115 °N, 115.5178°E)	forest	SD18B02	*Beauveria* sp2	PP863322	PV659042	PV659047	PV659052	
					ITS	*LSU*			
JX20	Guangfeng District, Shangrao City (28.3824°N, 118.2482°E)	forest	JX20B02	*Gongronella* sp1	PP907791	PP495838			
JX09	Yifeng County, Yichun City (28.3891°N, 114.7672°E)	crop	JX09A02	*Gongronella* sp2	PP905125	PP495827			
JX15	Ningdu County, Ganzhou City (26.9168°N, 116.0076°E)	forest	JX15B03	*Gongronella* sp2	PV112675	PP495828			
					ITS	LSU	*tef1*	*RPB2*	*act1*
JS06	Danyang City, Zhenjiang City (32.0632 °N, 119.5066°E)	grass	JS06C01	*Sarocladium* sp1	PP863316	PP930917	PV294972	PV294971	PV294970
JS07	Liyang City, Changzhou City (31.5736 °N, 119.3861°E)	grass	JS07C02	*Sarocladium* sp1	PP863317	PV248878	PV294975	PV294974	PV294973
JX12	Poyang County, Shangrao City (29.0714°N, 116.7042°E)	fallow	JX12E02	*Sarocladium* sp2	PP863315	PP930916	PV294978	PV294977	PV294976

ZJ19	Fuyang District, Hangzhou City (30.1530°N, 119.9847°E)	crop	ZJ19A01	*Sarocladium* sp2	PP863314	PV248877	PV294981	PV294980	PV294979
					ITS	LSU	*tef1*	*β-tub*	
JX11	Pengze County, Jiujiang City (29.7943° N, 116.5754°E)	forest	JX11B02	*Yunnania* sp	PP892051	PP930918	PV131306	PV131307	
	**total strain number**		**455**						

3	*Asp. aureoterreus*
9	*Asp. flavus*
2	*Asp. fumigatus*
4	*Asp. insuetus*
3	*Asp. ochraceus*
8	*Bea. bassiana*
6	*Cha. cochliodes*
2	*Cha. globosum*
8	*Clo. chloroleuca*
5	*Fus. solani*
2	*Lec. anqingense*
2	*Lec. renii*
13	*Mar. marquandii*
29	*Met. anisopliae*
3	*Met. baoshanense*
3	*Met. brunneum*
1	*Met. bulbillosa*
3	*Met. gaoligongense*
18	*Met. robertsii*
6	*Pen. brefeldianum*
65	*Pen. citrinum*
4	*Pen. daleae*
4	*Pen. guanacastense*
10	*Pen. janthinellum*
1	*Pen. oxalicum*
1	*Pen. sizovae*
8	*Ple. cucumerina*
1	*Poc. chlamydosporia*
1	*Pur. jiangxiense*
10	*Pur. lavendulum*
190	*Pur. lilacinum*
1	*Sim. subtropicum*
6	*Tri. asperellum*
1	*Tri. citrinoviride*
11	*Tri. spirale*
1	*Beauveria* sp1
2	*Beauveria* sp2
1	*Gongronella* sp1
2	*Gongronella* sp2
2	*Sarocladium* sp1
2	*Sarocladium* sp2
1	*Yunnania* sp
455	
